# Microengineered devices enable long-term imaging of the ventral nerve cord in behaving adult *Drosophila*

**DOI:** 10.1038/s41467-022-32571-y

**Published:** 2022-08-25

**Authors:** Laura Hermans, Murat Kaynak, Jonas Braun, Victor Lobato Ríos, Chin-Lin Chen, Adam Friedberg, Semih Günel, Florian Aymanns, Mahmut Selman Sakar, Pavan Ramdya

**Affiliations:** 1 Neuroengineering Laboratory, Brain Mind Institute & Institute of Bioengineering, EPFL, Lausanne, Switzerland; 2 Microbiorobotic Systems Laboratory, Institute of Mechanical Engineering & Institute of Bioengineering, EPFL, Lausanne, Switzerland; 3 Computer Vision Laboratory, EPFL, Lausanne, Switzerland

**Keywords:** Spinal cord, Fluorescence imaging, Spinal cord injury, Neural circuits

## Abstract

The dynamics and connectivity of neural circuits continuously change on timescales ranging from milliseconds to an animal’s lifetime. Therefore, to understand biological networks, minimally invasive methods are required to repeatedly record them in behaving animals. Here we describe a suite of devices that enable long-term optical recordings of the adult *Drosophila melanogaster* ventral nerve cord (VNC). These consist of transparent, numbered windows to replace thoracic exoskeleton, compliant implants to displace internal organs, a precision arm to assist implantation, and a hinged stage to repeatedly tether flies. To validate and illustrate our toolkit we (i) show minimal impact on animal behavior and survival, (ii) follow the degradation of chordotonal organ mechanosensory nerve terminals over weeks after leg amputation, and (iii) uncover waves of neural activity caffeine ingestion. Thus, our long-term imaging toolkit opens up the investigation of premotor and motor circuit adaptations in response to injury, drug ingestion, aging, learning, and disease.

## Abstract (web summary)

Minimally invasive procedures for tracking neural activity are important for understanding of neural networks. Here the authors describe microfabricated implants and windows that enable long-term recordings of motor circuit activity in Drosophila, allowing them to watch how neurons change their structure and activity over weeks.

## Introduction

Neural tissues are remarkably plastic, adapting to changes in internal states and in response to repeated exposure to salient environmental cues. In neuroscience, physiological studies of long timescale phenomena, including memory formation and neurodegeneration, have often relied upon comparing data pooled across animals sampled at multiple time points. However, quantifying differences across conditions with this approach suffers from inter-individual variability. Thus, longitudinal recordings of the same animal would be ideal to uncover adaptive changes in the functional and structural dynamics of neural circuits. Important technical challenges must be overcome to perform long-term studies in individual animals, including the minimization of experimental insults.

With the advent of microscopy-based neural recordings most notably two-photon calcium imaging^1^ it has become possible to chronically record brain circuits in vivo in a minimally invasive manner by leveraging chronic devices. For example, cranial window technologies were first developed to study mouse neocortex^2^ and have since been improved to acquire larger^3^ and deeper ^4^ imaging fields-of-view, as well as longer duration recordings^5^. Similar to rodents, brain imaging can also be performed in the behaving adult fly, *Drosophila melanogaster*^6,7^, a popular model organism that is (i) genetically tractable, (ii) has a small nervous system with many fewer neurons than rodents, and (iii) generates complex social, navigation, and motor behaviors^8–11^.

Recent approaches have enabled long-term chronic recordings of neurons in the fly brain^12–14^. Similar to imaging the neocortex of rodents with a cranial window^15^, the fly brain can be made optically accessible by removing head capsule cuticle and underlying tissues^6^. To perform long-term or repeated imaging, this hole can then be covered with UV-curable glue^13^, two-component silicon^16^, or manually-cut coverglass^12^. However, the techniques and technologies used to perform long-term imaging in the brains of mice and flies are not suitable to record motor circuits in the mammalian spinal cord, or insect ventral nerve cord (VNC). Like the spinal cord, which is obscured by vertebral bone, muscle, and dorsal lamina^17^, optical access to the VNC requires the removal of multiple overlying organs and tissues including the flight muscles, fat bodies, gut, and trachea. Invasive surgeries on the spinal cord permit the implantation of a chamber^18^, or a clamp^19^. However, the small size of the fly limits the use of conventional implantable devices, representing an important challenge to uncovering general principles for motor control through the investigation of the experimentally tractable VNC—a nervous tissue that is coarsely organized like the mammalian spinal cord^20^, and whose control principles resemble those found in vertebrates^21,22^.

We recently developed a dissection approach that gives optical access to the VNC in tethered, behaving animals by surgically and genetically—in the case of the indirect flight muscles—removing overlying tissues^23^. However, this technique is invasive, requiring the resection of thoracic organs and leaving open the thoracic cavity during imaging. This precludes recordings that last beyond a few hours. Consequently, it has been impossible to perform repeated measurements of premotor and motor circuits in the same fly to shed light on how these circuits adapt over time. Existing tools for long-term brain imaging are insufficient for long-term VNC imaging. Unlike for brain imaging, one cannot glue^13^, or manually cut coverslips^12^ to cover the thoracic hole following dissection. Instead, one needs a more precise, reproducible approach that ensures that hemolymph does not leak from the thorax. Additionally, to gain optical access to the VNC one must gently but firmly displace large overlying thoracic organs and tissues without disrupting their function.

Here, we describe a suite of microengineered devices that address all of these challenges to permit long-term and repeated recordings of the *Drosophila* VNC for more than one month. These devices address the unique challenges associated with studying small model organisms (the fly is ~2–3 mm long) that require extremely gentle manipulation. Specifically, we designed (i) a manipulator (‘arm’) that allows us to move aside and temporarily hold in place thoracic organs, (ii) flexible implants that eliminate the need to surgically remove thoracic organs to gain optical access to the VNC, (iii) a transparent polymer window that encloses the thoracic cavity and is numbered to allow individual flies to be distinguished from one another across imaging sessions, and (iv) a remounting stage for gently yet firmly tethering flies for repeated recordings. We provide detailed descriptions of how to fabricate and use all of these tools to facilitate their replication and adoption by other laboratories.

We demonstrate that implants and windows have minimal impact on animal survival and locomotion, and that they permit neural recordings across at least one month. Then we illustrate use-cases of our long-term imaging toolkit in two proof-of-concept studies. First, we longitudinally measure the degradation of limb mechanosensory neuron innervation of the VNC during two weeks following leg removal. Second, we illustrate how—by leaving thoracic organs intact—one can measure the impact of drug ingestion on neural population dynamics. Thus, our long-term thoracic imaging toolkit enables the repeated, longitudinal study of structural and functional neural dynamics in premotor and motor circuits. These tools may more generally also be used to study other thoracic tissues including indirect flight muscles, gut, and trachea.

## Results

### Long-term recording toolkit and experimental workflow

**Fig. 1:**
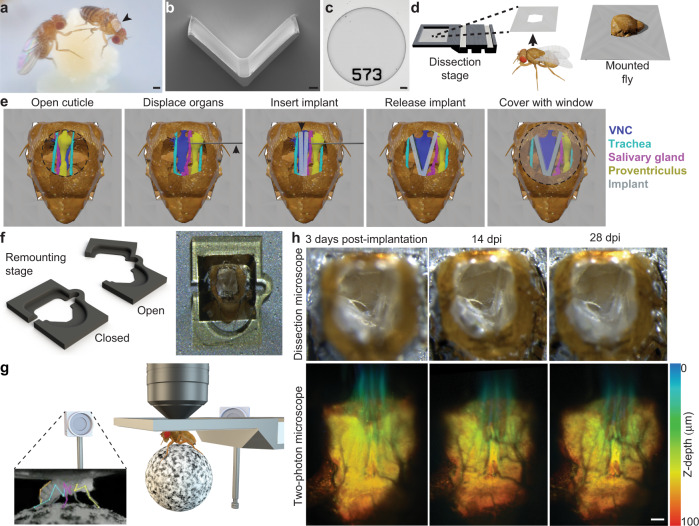
Long-term recording technologies, workflow, and experimental validation. **a** Implanted adult flies can be raised in complex environments. An implanted animal---see dorsal thoracic window (black arrow)---interacts with a non-implanted animal. Scale bar is 0.5 mm. **b** A mechanically compliant, transparent implant microfabricated from Ostemer 220. Scale bar is 50 μm. **c** A numbered, transparent thoracic window microfabricated from SU-8. Scale bar is 50 μm. **d** For implantation, an animal is mounted, thorax first, into a hole in a steel shim within a dissection stage. **e** A multi-step dissection permits long-term optical access to the ventral nerve cord (VNC). **left** A hole is cut into the dorsal thoracic cuticle, revealing the proventriculus (yellow), trachea (cyan), and salivary gland (magenta) overlying the ventral nerve cord (VNC, dark blue). The indirect flight muscles (IFMs) were degraded by tissue-specific expression of Reaper (*Act88F:Rpr*)^23^. ﻿Then, using a custom-designed manipulator arm, thoracic organs are displaced, revealing the VNC. **middle** Next, the implant is placed within this thoracic hole in a narrow, mechanically closed configuration. **right** T﻿he arm is removed and the implant is released, causing it to open and mechanically push aside organs covering the VNC. Finally, a transparent window is sealed to enclose the thoracic hole. **f** A 3D nanoprinted remounting stage permits mounting and dismounting of animals for repeated two-photon imaging. **left** The monolithic mechanism is actuated around a compliant hinge. **right** Sample image of an animal tethered to the remounting stage, as seen from above. **g** Implanted animals tethered to the remounting stage are placed under a two-photon microscope surrounded by a camera array. This configuration permits simultaneous recordings of neural activity and animal behavior. Inset shows one camera image superimposed by deep learning-based 2D poses estimated using DeepFly3D^25^. **h** (top row) The dorsal thorax of an implanted animal, seen from the dissection microscope, and (bottom row) its VNC, visualized using two-photon microscopy. This animal expresses GFP throughout the nervous system and is recorded at **left** 3 dpi, **middle** 14 dpi, and **right** 28 dpi. Z-stacks are depth color-coded (100 μm). Scale bar is 25 μm.

We developed microengineered devices and associated micromanipulation protocols that enable optical access to the fly’s VNC for more than one month. Implanted flies exhibit no obvious deficits in their ability to feed, walk, lay eggs, or interact with others. (Fig. 1a and Supplementary Movie 1). Optical access depends on two main components: a compliant and transparent implant (Fig. 1b) and a transparent thoracic window (Fig. 1c). The fabrication of windows must be reproducible and they must be large enough to securely seal the thoracic opening to prevent hemolymph leakage. Solutions developed for long-term brain imaging such as sealing with UV-curable glue^13^ or a manually cut piece of coverglass^12^ do not address these challenges. We designed and microfabricated a custom window using a biocompatible polymer, SU-8, through photolithography (Supplementary Fig. 1). Because implants are not required for long-term brain imaging, these had to be designed *de novo* and without inspiration from previous approaches. Our earliest implants were rigid structures intended to protect VNC imaging regions-of-interest (ROIs) from occlusion by thoracic tissues (Supplementary Fig. 2, ‘iterations 1 and 2’). Later iterations attempted to combine thoracic windows with protective pillars (Supplementary Fig. 2, ‘iterations 3 and 4’). However, both of these initial prototypes yielded prohibitively low survival rates. We achieved a breakthrough by taking a completely different approach: we designed compliant (polymer-based), mechanically compressible V-shaped implants. Through several iterations and testing, we converged upon the specific size and angle of these implants resulting in a very high post-implantation survival rate. We fabricated these implants *en masse* using soft lithography, a technique that is based on rapid prototyping and replica molding (Supplementary Figs. 3 and 4).

To use these tools, we developed a manipulation protocol illustrated in Supplementary Movie 2. Briefly, we first mount animals onto a surgical dissection stage using UV-curable glue (Fig. 1d)^23^. Next, we cut a square-shaped hole into the dorsal cuticle using a 30G syringe needle (Fig. 1e - left panel). The indirect flight muscles (IFMs) are subsequently removed to create a thoracic opening for the implant. To minimize the impact of the microsurgery, we work with animals expressing the apoptosis-inducing protein, Reaper, specifically in IFMs (*Act88F:Rpr*). Expressing Reaper results in rapid degradation of the muscle tissue^23^, the remainder of which can easily be removed with the syringe needle. However, this genetic line is not strictly required to make use of these tools. Having exposed the thoracic tissues, we then use a fine glass needle and forceps to unilaterally detach tracheal fibers that connect the gut and left salivary gland. We designed a custom manipulation arm (Supplementary Fig. 5) to push internal organs—gut, salivary gland and trachea—to the right side of the thoracic cavity (Fig. 1e - left panel). This allows one to insert the implant in a closed state into the newly accessible thoracic cavity (Fig. 1e - middle panel and Supplementary Fig. 4). Upon release, the implant gradually opens holding the organs against the thoracic wall after the manipulation arm is retracted (Fig. 1e - right panel). We seal the exposed thoracic cavity by gluing a transparent polymer window to the cuticle (Fig. 1e - right panel). These windows have unique numbers engraved on their surfaces which make it possible to identify and distinguish between implanted animals. We can then detach animals by removing the UV-curable glue holding the scutellum to the dissection stage, allowing them to behave freely.

To facilitate repeated, gentle tethering of implanted flies we printed a remounting stage (Fig. 1f and Supplementary Fig. 6) using two-photon polymerization^24^. This manufacturing process has the accuracy required to fabricate 3D features that reliably hold animals in place. When mounted, we studied animals using a two-photon microscope system surrounded by a multi-camera array. This system enables simultaneous recordings of neural activity in the VNC^23^ as well as markerless 3D body part tracking^25^ (Fig. 1g). In the vast majority of cases our implantation protocol was successful. Infrequently, implanted animals exhibited specific movements of respiratory, or digestive tissues that could occlude the VNC during imaging (Supplementary Fig. 7). Successful implantation permitted optical access to the VNC that remained largely unchanged over one month. This allowed repeated studies of the structure (Fig. 1h and Supplementary Movie 3) and functional dynamics of neural populations in female (Supplementary Movie 4) and male (Supplementary Movie 5) flies. Our long-term recording tools could also be applied to recording genetically-identifiable pairs of neurons. We illustrate this by imaging the activity of a pair of DNa01 descending neurons over three days---1, 3, and 5 days-post-implantation (dpi)). This activity correlates with locomotor turning^23^ (Supplementary Fig. 8 and Supplementary Movie 6). Although here we focused on gaining optical access to the VNC’s cervical connective and prothoracic (T1) neuromere (Fig. 1h), our approach is general, permitting the redesign and microfabrication of implants that enable the imaging of other regions of the VNC. As a proof-of-concept, we modified an implant, making it possible to restrain tissue in the posterior thorax and to gain optical access to T2 and T3 neuromeres (Supplementary Fig. 9).

### Impact of long-term imaging technologies on lifespan and behavior

**Fig. 2:**
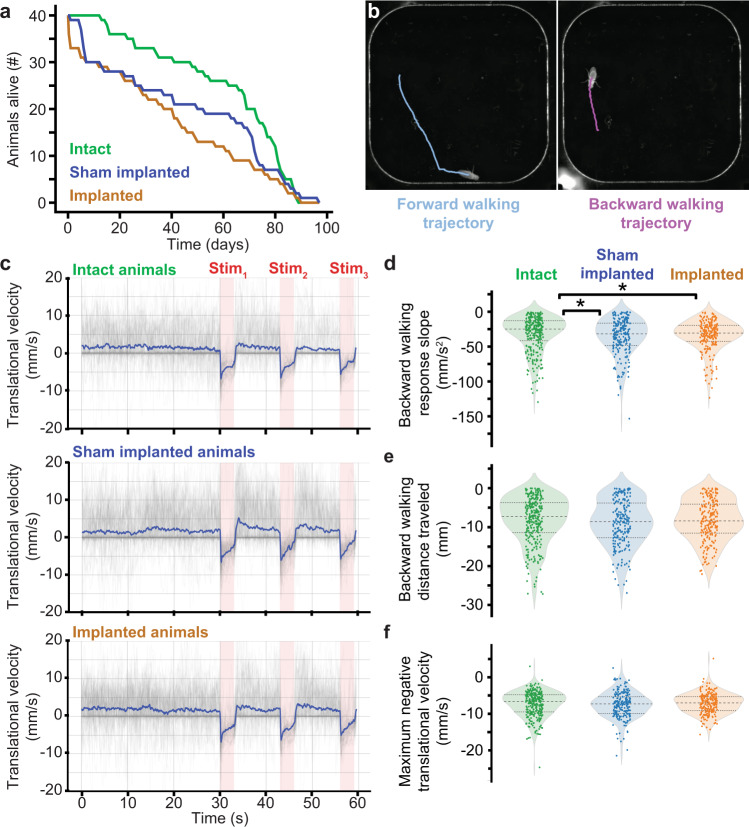
Impact of long-term imaging technologies on lifespan and behavior. **a** Survival curves for genetically-identical sibling animals that were (i) not experimentally manipulated (green, `Intact'), (ii) tethered, cold anaesthetized, and had their wings removed (blue, `Sham implanted'), or (iii) prepared for long-term imaging by implantation and the addition of a thoracic window (orange, `Implanted'). Source data are provided as a Source Data file. **b** Behaviors were compared by analyzing the dynamics of optogenetically activated backward walking within a rounded square-shaped arena. Locomotion was computationally analyzed and plotted, showing the animal's initial forward trajectory (cyan) and subsequent optically evoked backward walking trajectory (purple). **c** Translational velocities of intact (top), sham implanted (middle), and implanted (bottom) animals during 30 s of spontaneous behavior, followed by three optogenetic stimulation periods of 3 s each (pink, `Stim'). Here, we pooled data recorded at three time periods over one month. Shown are raw (gray) and mean (blue) traces. From these time-series, each stimulation event is one data point from which we calculated summary statistics (intact group, *n* = 286 events; sham implanted group, *n* = 208 events; implanted group, *n* = 213 events) including (**d**) the initial negative slope in translational velocity---backward walking---upon optogenetic stimulation, (**e**) the backward walking distance traveled over the entire optogenetic stimulation period, and (**f**) the peak negative translational velocity over the entire optogenetic stimulation period. One asterisk (*) indicates *P* < 0.05. Source data are provided as a Source Data file.

To validate our long-term recording approach, we first studied the potential impact of implantation on lifespan. Specifically, we measured the longevity of three groups of animals (*n* = 40 per group): (i) flies that were not manipulated (‘Intact’), (ii) animals that endured cold anesthesia, mounting onto the dissection stage, and wing removal (‘Sham implanted’), or (iii) flies that underwent the full implantation procedure (‘Implanted’). For this experiment, 73% of implanted animals survived more than 4 h following surgery. Because the surgical and implantation procedure for a single fly takes ~40 min from start to finish total throughput—a combination of this implantation time and acute survival rate—was one implanted animal per 55 min. Among flies that survived more than 4 h after implantation, we observed a maximum survival of up to 88 days. However, implanted flies exhibited higher mortality than intact animals in the first days following implantation (Fig. 2a). Notably, sham implanted flies had similarly increased mortality in the first days, suggesting that pre-implantation animal handling, and not implantation itself, was responsible for increased mortality. We obtained similar survival rates for implanted versus intact male flies examined over 20 days (Supplementary Fig. 10a).

Next, although implantation did not dramatically affect longevity, we reasoned that placing a microfabricated object within the thorax might negatively impact walking, possibly due to the perturbation of leg-related musculature, or simply the additional load. Investigating this possibility is difficult because flies use a variety of diverse gaits at different walking speeds and maneuvers^26^. Therefore, to be able to perform a quantitative analysis of walking, we drove stereotypical backward walking by optogenetic activation of the light-gated cation channel, CsChrimson^27^ expressed in Moonwalker Descending Neurons (MDNs)^28,29^. We stimulated animals in a custom-built arena (Fig. 2b) repeatedly over the course of one month. This allowed us to quantify and compare the walking trajectories of intact, sham implanted, or implanted flies. We first recorded spontaneous behaviors for 30 s, and then delivered three consecutive flashes of 590 nm orange light for 3 s each (Fig. 2c, pink and Supplementary Fig. 11a) with an inter-stimulus interval of 10 s. These experiments were performed on the same animals at three periods of time over one month (1–3 days post implantation (dpi), 14–16 dpi, and 28–30 dpi). Upon optogenetic stimulation, we observed that animals generated fast backward walking that gradually slowed and rapidly returned to baseline when the light was turned off (Supplementary Movie 7). Over all recording sessions we observed a small significant difference in the initial backward acceleration (Fig. 2d) between the intact group and the sham implanted group (*P* = 0.044 Kruskal–Wallis with post-hoc Conover test) and between the ’intact’ group and the ’implanted’ group (*P* = 0.044). However, no statistical difference were observed in the translational velocities of intact, sham implanted, and implanted animals in terms of the total backward walking distance traveled (Fig. 2e), and the maximum backward walking velocity (Fig. 2f). Similar results were obtained when comparing age-restricted cohorts. Specifically, we did not observe a difference between the intact and implanted groups in the initial backward acceleration for any age cohort (1–3 dpi, 14–16 dpi, or 28–30 dpi). Intact flies had a slightly slower backward acceleration (this may be because of contact between the legs and wings that were sagging in Act88F:Rpr expressing animals). We, noted a significant difference between both control groups at 14–16 dpi (Supplementary Fig. 11b, c) for (i) the backward walking response slope (*P* = 0.009), (ii) the backward walking distance traveled (*P* = 0.0008), and (iii) the maximum negative translational velocity (*P* = 0.003). Spontaneous walking was also qualitatively unchanged in male implanted versus intact flies (Supplementary Movie 8). Taken together, these results suggest that locomotion is not significantly affected by implantation.

### Quantifying long-term nerve degradation in the VNC following limb amputation

**Fig. 3:**
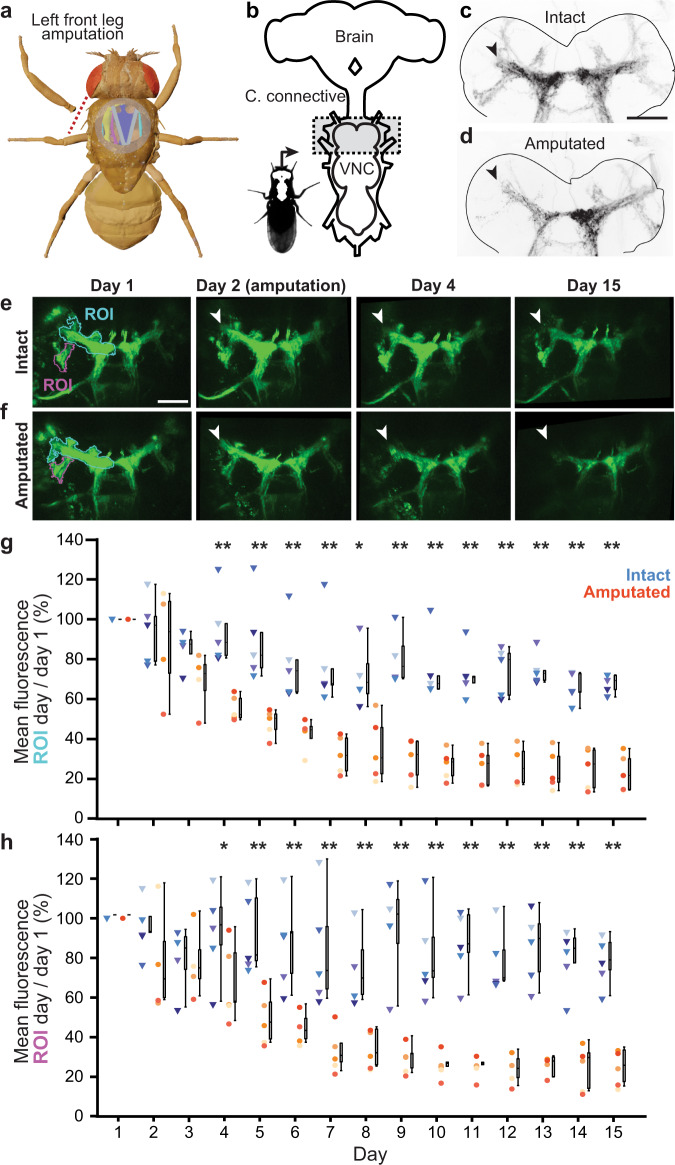
Long-term imaging of mechanosensory nerve degradation in the VNC following leg amputation. **a** In experimental animals the front left leg was amputated at the thorax-coxa joint at 2 dpi. **b** Fly nervous system schematic. The VNC region imaged is highlighted (gray). Standard deviation z-projection of confocal imaging volumes recorded from flies that were either (**c**) left intact, or (**d**) amputated at 2 dpi (nc82 stained neuropil outlined in gray; GFP fluorescence in black). Tissues were taken from implanted animals whose front left legs were amputated. VNC tissue was removed and stained at 20 dpi. Black arrowhead indicates VNC region exhibiting greatest difference between intact and amputated proprioceptor innervation. Scale bar is 50 μm. Maximum intensity projections of z-stacks recorded from (**e**) an intact (control), or (**f**) amputated animal. Data were acquired using two-photon microscopy. Shown are images taken at 1, 2, 4, and 15 dpi. Images are registered to the 1 dpi image. Scale bar is 50 μm. White arrowheads indicate degrading axon terminals in the VNC of amputated animals. **g**, **h** Fluorescence measured across days using two-photon microscopy from intact (*n* = 5; blue triangles), or amputated animals (*n* = 5; orange-red circles). Measurements indicate mean fluorescence within the (**g**) cyan, or (**h**) purple region-of-interest (ROI) as in panels **e** and **f**, normalized and divided by the mean fluorescence at 1 dpi. Box plots indicate median, first and third quartiles. Statistical comparisons performed with a Mann–Whitney *U* Test (two-sided) for which one asterisk (*) indicates *P* < 0.05 and two asterisks (**) indicate *P* < 0.01. For panel **g**, *P* = 0.006 (day 4); 0.006 (day 5); 0.009 (day 6); 0.006 (day 7); 0.018 (day 8); 0.009 (day 9); 0.006 (day 10); 0.006 (day 11); 0.009 (day 12); 0.006 (day 13); 0.006 (day 14); 0.006 (day 15). For panel **h**, *P* = 0.03 (day 4); 0.006 (day 5); 0.009 (day 6); 0.006 (day 7); 0.009 (day 8); 0.009 (day 9); 0.006 (day 10); 0.006 (day 11); 0.009 (day 12); 0.006 (day 13); 0.006 (day 14); 0.006 (day 15). Source data are provided as a Source Data file.

Neuronal circuits retain a capacity for structural rearrangement throughout adulthood^30,31^. This enables adaptations in response to nervous system injury^32–34^, and stroke^35^. Similarly, in flies, locomotor gaits reorganize following leg amputation^36^. However, the impact of amputation on limb control circuits remains unexplored because uncovering these changes requires imaging the VNC of amputated animals across days or weeks. Although, in principle, one could instead pool data across multiple animals at specific days following leg amputation, this has two major shortcomings. First, inter-animal variability would limit the ability to resolve the degeneration of specific neural structures. Second, this approach would be more time consuming, requiring many more experiments and animals per timepoint of interest. By contrast, longitudinal imaging is ideally suited for uncovering dynamic changes in neuronal structures using relatively few animals. To illustrate this possibility we followed the degradation of primary proprioceptive mechanosensory afferents in the VNC after leg amputation. Specifically, we visualized the axon terminals of amputated proprioceptive leg femoral chordotonal organs (*Act88F-Rpr/+; iav-Gal4/UAS-GFP; +/+*) in the T1 neuromere of the VNC. Flies were implanted on the first day post-eclosion (dpe). Then, one day post-implantation (1 dpi) we performed volumetric two-photon imaging of T1. Volumes consisted of 100 images taken at 1 *μ*m depth intervals. Then, at 2 dpi, the front left leg of each experimental animal was amputated near the thorax-coxa joint (Fig. 3a).

Implanted flies tolerated leg amputation and generated normal behaviors with the remaining five legs (as in ref. 36). Every day for 15 days, we collected T1 image volumes (Fig. 3b) from control animals (‘Intact’), and from animals with their front left legs removed (‘Amputated’). We performed VNC dissection, staining, and confocal imaging 18 days after leg removal to confirm that mechanosensory innervation of the VNC’s T1 neuromere persisted in intact animals (Fig. 3c) but degraded in amputated animals (Fig. 3d). Close examination of two-photon microscope image volumes revealed that, in control animals, some photobleaching occurred in the imaging region over days (Fig. 3e). However, this decline in fluorescence was not nearly as profound as the reduction in signal observed in chordotonal organ axon terminals of the left T1 neuropil of amputated animals (Fig. 3f; Supplementary Fig. 12 and Supplementary Movie 9). By quantifying changes in signal intensity within specific regions of interest (ROIs) of chordotonal axon innervations within the VNC ^37^, we measured a highly reproducible and significant fluorescence reduction in amputated versus intact animals (Mann–Whitney *U* Test, * indicates *P* < 0.05 and ** indicates *P* < 0.01) (Fig. 3g, h).

### Capturing neural population dynamics associated with drug ingestion

In addition to being morphologically adaptable across days and weeks, neural circuits also continuously modulate their dynamics on shorter timescales depending on the internal state of an animal. In *Drosophila*, as in vertebrates, these states include hunger^38^, fatigue^39^, sexual arousal^40^, aggression^41^, and defensive arousal^42^. Internal states can also change following the ingestion of psychoactive substances including caffeine^43–45^. Continuous monitoring of the nervous system is crucial to uncover how circuits reconfigure during these changing states.

Our previous technique for studying VNC neural dynamics in behaving animals^23^ required the removal of large sections of gut, reducing the longevity of animals and making long-term recordings that capture changes in internal states impossible. Furthermore, although taste responses can be studied in the brain^46^, removing the gut precludes feeding, and, consequently, does not allow one to investigate how satiety, or the ingestion of psychoactive substances modulate neural dynamics in the VNC. Our long-term imaging toolkit preserves the gut, making it possible for animals to be fed during two-photon microscopy. Therefore, we next asked to what extent our technologies could be used to uncover the impact of drug ingestion on neural dynamics.

**Fig. 4:**
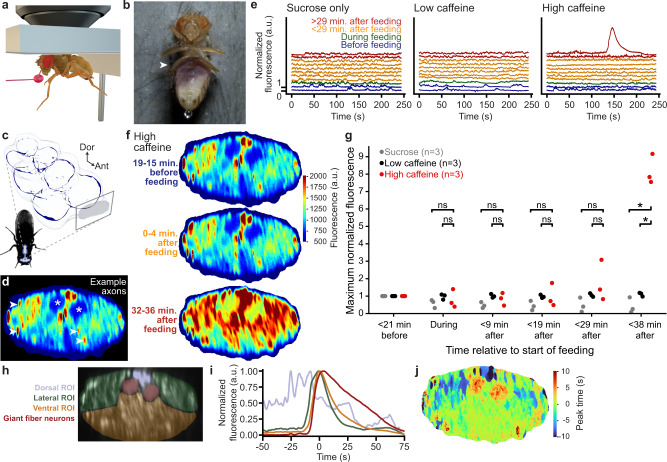
Continuous imaging of neural population dynamics before, during, and after ingestion. **a** Digital rendering of a fly being fed while neurons are recorded using a two-photon microscope. **b** Photo of an implanted animal after ingesting a high-concentration caffeine solution during two-photon microscopy. White arrowhead indicates purple coloration of the abdomen, confirming digestion of a caffeine-sucrose solution mixed with Amaranth dye. **c** Schematic of the ventral nerve cord indicating the imaging region in the neck connective. **d** Color-coded mean neural activity during all non-locomotor periods for a fly before feeding (same data as top panel in **f**) including annotations of individual axons passing through the neck connective (arrowheads). Asterisks indicate location of giant fiber axons. **e** Normalized fluorescence across all axons passing through the thoracic neck connective during 4 min recordings either before (blue), during (green), soon after (orange), or long after (red) feeding. Flies were fed with a solution containing either only sucrose (left), sucrose and a low-dose (middle), or high-dose of caffeine (right). **f** Color-coded mean neural activity during all non-locomotor periods for a fly either before (top), immediately after (middle), or long after (bottom) ingestion of a high-concentration caffeine solution. **g** Statistical analysis indicating the presence of activity waves. Neural activity is normalized using parameters computed on pre-feeding activity (Methods). Maximum normalized activity is shown for three flies per condition before, during, and after feeding. Maximum activity is only significantly increased > 29 min after feeding with a high concentration caffeine solution (one-sided Mann–Whitney *U* tests, * indicates *P* < 0.05, *P* = 0.04 for both * reported, ns indicates not significant). Source data are provided as a Source Data file. **h** The cervical connective in one implanted animal is segmented into four regions-of-interest (ROIs). These are overlaid onto a standard-deviation time-projection image. **i** Neural activity normalized to peak fluorescence during a wave of activity. Traces are color-coded as in panel **h**. The peak of mean fluorescence across all regions is centered on 0 s. **j** Pixel-wise time of peak activity. The peak of mean activity across the entire neck connective set as 0 s.

Flies exposed to low doses of caffeine have reduced sleep^43,44^ and increased locomotor activity^45^. Here, we asked to what extent caffeine ingestion might also drive global changes in neural population dynamics. To test this, we first starved animals for 21–23 h to encourage feeding. Then, after implantation, we recorded neural activity in the cervical connective (‘Before feeding’). While continuing to record neural activity, animals were then fed (Fig. 4a) either a control solution (‘Sucrose only’) containing 8 mg/ml sucrose and 1 mg amaranth dye (to confirm feeding^47^) (Supplementary Movie 10), or an experimental solution that also contained 8 mg/ml, or 40 mg/ml caffeine: ‘Low caffeine’ (Supplementary Movie 11), or ‘High caffeine’ (Supplementary Movie 12), respectively. We continued to record neural activity and behavior for the next 38 min. Feeding was confirmed by post-hoc evaluation of abdominal coloration from dye ingestion (Fig. 4b). During imaging, we examined the activity of ascending and descending neurons whose axons pass through the cervical connective linking the brain and VNC. To do this, we performed coronal cross-section two-photon imaging of the thoracic cervical connective (Fig. 4c)^23^ in flies expressing the genetically encoded calcium indicator, GCaMP6f, as well as the anatomical marker, tdTomato, throughout the nervous system (*Act88F-Rpr/+; GMR57C10-Gal4/UAS-opGCaMP6f; UAS-tdTomato/+*). To overcome image deformation and translation occurring during animal behaviors, we registered imaging data using an optic flow-based algorithm (see Methods)^23^. After image registration, our coronal imaging data reveals the activity of axons belonging to descending neurons that drive actions^48,49^, and ascending neurons that convey ongoing behavioral states to the brain^50^. These axons are apparent as ellipses in our two-photon microscopy images (Fig. 4d, white arrowheads). These pan-neuronal regions-of-interest (ROIs) are consistent with those we observed when imaging neural activity in very sparse sets of descending^23^ and ascending^50^ neurons. This is most apparent for the pair of large Giant Fiber descending neuron axons^51^ that pass through the dorsal midline of the cervical connective (Fig. 4d, white asterisks).

Across all three experimental conditions—before, during, and shortly after feeding—we observed fluctuations in neural activity that were associated with epochs of walking (Fig. 4e, blue, green, and orange traces; Supplementary Fig. 13c, Fly 7 and Supplementary Movies 10–12). However, more than 29 min after feeding, we observed large waves of activity uniquely in flies fed the high concentration caffeine solution (Fig. 4e, red traces). Waves were much larger in amplitude than neural activity fluctuations associated with spontaneous locomotion. Activity waves spread across the entire connective (Fig. 4f) and were associated with an overall rigid pose accompanied by micromovements (Supplementary Movie 13). To quantify the presence and amplitude of waves at different time points and between experimental conditions, we normalized neural activity across the cervical connective relative to that observed before feeding for each fly. We then computed the maximum normalized fluorescence (upper bound of the 99% percentile) for periods before, during and after feeding. Up to ~29 min after feeding, the maximum activity level of flies fed with a high concentration of caffeine was not significantly different from flies fed with sucrose, or a low caffeine solution (Mann–Whitney *U* tests, *P* > 0.05). By contrast, between 29 and 38 min after feeding, the maximum activity of each fly fed with high caffeine solution was significantly higher than the other conditions (Mann–Whitney *U* tests, *P* = 0.040), due to the wave of neural activity (Fig. 4g). The temporal evolution of these waves was also reproducible: activity began in the dorsalmedial (blue), then dorsolateral (green), and then ventral (orange) connective. The Giant Fiber neurons (red)^51^ were last to become active and sustained high activity for longer periods of time (Fig. 4h–j and Supplementary Fig. 13d–i). These data illustrate that our long-term imaging toolkit can be used to investigate how food or drug ingestion influences internal states and global neural dynamics.

## Discussion

Here we have described microengineered devices that open up the comprehensive and longitudinal investigation of motor control in behaving animals. Specifically, we enable long-term two-photon recordings of tissues in the adult *Drosophila* thorax including premotor, sensory, and motor circuits targeting and residing within the VNC. Our toolkit is designed to address the unique challenges of long-term recording of the VNC as opposed to simpler approaches that enable longitudinal recordings of neurons in the brain^12,13^: it consists of (i) a micromanipulator arm, (ii) a polymer-based soft implant for displacing thoracic organs, (iii) a numbered, transparent polymer window that can be fabricated in a reproducible manner to seal the thoracic opening, and (iv) a compliant tethering stage that permits gentle, repeated mounting around the thorax of animals for two-photon imaging. Together, these tools expand the neural recording time window from only a few hours^23^ to more than one month, without markedly reducing the lifespan of implanted animals or significantly perturbing their locomotor behavior. We have illustrated several use cases for our long-term imaging approach including (i) weeks-long recordings of neural morphology, pan-neuronal activity, and sparse neural activity, (ii) weeks-long degradation of limb sensory neuron projections to the VNC from an amputated limb, and (iii) global changes in neural activity following drug ingestion.

Our longevity experiments confirmed that the lifespans of implanted flies was similar to those of intact animals. The survival curves were, however, shifted for implanted and sham implanted flies due to excess mortality within the first few days following surgery. This suggests that those initial losses might be due to surgical handling and not specifically linked to implantation. Consistent with this, our studies of backward walking revealed no clear changes in locomotor metrics between control and implanted animals. However, in the future, it would be important to confirm the minimal impact of implantation on more complex behaviors like courtship and gap-crossing.

In a first case study of our toolkit, we investigated the anatomical degradation of chordotonal projections to the VNC over two weeks following leg amputation. There we observed a marked degradation of mechanosensory neuron terminals in the first week following amputation. The time evolution of this degradation was heterogeneous across ROIs, consistent with the existence of distinct chordotonal cell populations ^37^ which may have varying levels of robustness to degradation. Alternatively, some terminals might arise via ascending projections from T2 (midleg) or T3 (hindleg) and thus are not directly affected by foreleg amputation. *Drosophila* has been previously used in numerous studies as a model of nerve injury^52^, where it has been shown that axonal degradation following injury can be similar to Wallerian degeneration in mammals^53^. Thus, our long-term imaging toolkit might in the future be used to test for pharmacological interventions that can slow down or prevent injury-induced axonal degeneration in motor circuits.

When analyzing the activity of descending and ascending neurons in the thoracic cervical connective following caffeine ingestion, we did not observe large changes in neural activity in the low-concentration caffeine condition, despite previously reported behavioral changes^45^. On the other hand, we observed large waves of neural activity following ingestion of a high-concentration caffeine solution. Some flies had several of these waves, suggesting that they are not due to calcium release from a terminal cell death process. However, the origin of these calcium dynamics remains unclear. For example, caffeine has been shown to induce cytoplasmic release of internal stores of Ca^2+^^54^. Although this may be unlikely in the case of caffeine ingestion, whether temporally propagating activity waves result from such a mechanism or profound neural firing remains unclear and could be the focus of future studies using these tools. Notably, we observed that animals did not recover after feeding on our high concentration caffeine solution. Nevertheless, this proof-of-concept illustrates how long-term imaging in *Drosophila* may be used in the future to screen for the impact of drug ingestion on neural dynamics in behaving animals.

Based on these successful case studies we envision that our microengineered long-term imaging devices can be leveraged to study a variety of additional questions and challenges. For example, one might apply these devices to record the progression of cell death in *Drosophila* models of neuronal disorders like Parkinson’s disease^55^. Our implant fabrication pipeline is also generalizable. Therefore, implant shapes could be adapted to address other experimental challenges including the targeting of abdominal ganglia circuits that regulate mating receptivity in females^56^. Furthermore, implants might be modified to store and release active components that, for example, deliver compounds into the hemolymph in a controlled manner. Finally, additional steps might be taken to automate implantation by, for example, opening the thoracic cuticle using a UV-excimer laser^57^, or developing robotic manipulation techniques to automatically displace thoracic organs, position the implant, and seal the thoracic hole with a window^58^.

In summary, the technological developments presented in this work permit a variety of experiments on individual flies across a wide range of time scales, opening up an understanding of how biological systems—in particular premotor and motor circuits—change during aging or disease progression, following injury, learning, or social experiences, in response to shifts in internal state, or as a consequence of food or drug ingestion. These data, in turn, can inspire the development of more adaptive controllers for artificial systems that have the capacity to shift in form and function to accommodate continuously changing capabilities and needs.

## Methods

### Fabrication of thoracic windows with engraved numbers

Thoracic windows (transparent polymer disks) were fabricated using photolithography^59^. All exposure steps were performed on a mask aligner (MJB4, Süss MicroTec, Germany) using i-line illumination. Chrome masks were fabricated using a direct laser writer (VPG-200, Heidelberg Instruments, Germany) and an automatic mask processor (HMR900, HamaTech, Germany). The dimensions of microfabricated structures were measured using an optical microscope (DM8000 M, Leica Microsystems, Switzerland) or a mechanical surface profiler (Dektak XT, Bruker Corporation, USA). The protocol began with treating the surface of a 4-inch silicon wafer with a plasma stripper (PVA TePla 300, PVA AG, Germany) at 500 W for 7 min to reduce its wettability. An aqueous solutions of 20% (wt/vol) Dextran (MP Biomedicals, MW 60k–90k g/mol) was spun at 1000 rpm (WS-650-23, Laurell Technologies Corporation, USA), and baked at 150 °C for 2 min to form a 1 μm thick water-soluble sacrificial layer. This layer permits windows to be gently released at the end of the fabrication process (Supplementary Fig. 1a-i). A negative photoresist (SU-8 3025, Kayaku Advanced Materials, USA) was directly spin-coated on the sacrificial layer and soft-baked (Supplementary Fig. 1a-ii). Notably, at this thickness, SU-8 has a transmittance of greater than 95%^60^ (https://kayakuam.com/wp-content/uploads/2020/07/KAM-SU-8-3000-Datasheet-7.10-final.pdf). After exposure, the windows were post-baked and uncured resist was removed with a developer (Propylene glycol methyl ether acetate (PGMEA, 1-methoxy-2-propanol acetate), Sigma-Aldrich, Germany) (Supplementary Fig. 1a-iii). Next, the wafer with SU-8 windows was coated with a 20 μm thick layer of positive photoresist (AZ 40XT) using an automated processing system (ACS200 Gen3, Süss MicroTec, Germany). This extra layer of polymer serves as a physical mask during the metal deposition process. A second chrome mask was fabricated to pattern unique identifiers onto the windows using photolithography. Next, the wafer was coated with Ti and Au films^61^ using physical vapor deposition (EVA 760, Alliance-Concept, France) at a thickness of 3 nm and 15 nm, respectively (Supplementary Fig. 1a-iv). The development of the negative photoresist (Remover 1165, Kayaku Adv. Mat., USA) removed all the layers on top of the windows except for the numbers that serve as markers. Finally, the labeled windows were released by dissolving the sacrificial layer in DI water (Supplementary Fig. 1a-vi). The windows were filtered, dried at room temperature, and sterilized prior to use in experiments. The resulting windows were optically transparent (Supplementary Fig. 1b) and of the appropriate size to seal thoracic openings (Supplementary Fig. 1c).

### Fabrication of polymer molds that are used to cast implants

We developed a two-level microfabrication technique to maximize throughput, protect master molds from excessive use, and facilitate technology dissemination^62,63^. Briefly, implants were cast within elastomer templates that were fabricated from an etched wafer serving as a master mold. First, a four-inch silicon test wafer (100/P/SS/01-100, Siegert Wafer, Germany) was treated with hexamethyldisilazane (HMDS) (CAS number: 999-97-3, Sigma-Aldrich, Germany) and dehydrated at 125 °C to enhance adhesion to its surface. The wafer was then spin-coated with an 8 μm thick film of positive photoresist (AZ 9260, Microchemicals GmbH, Germany) using an automatic resist processing system (EVG 150, EV Group, Germany)(Supplementary Fig. 3a-i). After baking, exposure, and development steps, the wafer was then processed using deep reactive ion etching (DRIE), specifically a Bosch process,^64^ (AMS 200 SE, Alcatel) to obtain nearly vertical walls with a high aspect ratio (Supplementary Fig. 3a-ii). The remaining positive resist was stripped in a remover (Remover 1165, Kayaku Advanced Materials, USA) at 70 °C and cleaned by rinsing with water and air drying (Supplementary Fig. 3a-iii). The elastomer templates were fabricated by replica molding using polydimethylsiloxane (PDMS). The replica molding process began with vapor deposition of silane (trichloro(1H,1H,2H,2H-perfluorooctyl) Silane, Sigma-Aldrich, Germany) onto the surface of the master mold in a vacuum chamber for 6 h. Silanizion was only performed once because it forms a permanent silane layer. PDMS was prepared as a mixture (10:1, wt/wt) of the elastomer and the curing agent (GMID number: 01673921, Dow Europe GmbH, Germany) and poured onto the wafer in a petri dish. To release any bubbles trapped inside the high aspect ratio wells, the mold was degassed using a vacuum pump (EV-A01-7, Swiss Vacuum Technologies SA, Switzerland) in a vacuum desiccator (F42020-0000, SP Bel-Art Labware & Apparatus, USA). Finally, the elastomer was cured at 65 °C for 5 h in an oven (UF30, Memmert GmbH, Germany) and the PDMS slab was peeled off (Supplementary Fig. 3b). Using alignment markers as a guide, the slab was then cut into several pieces with a razor blade to serve as templates with which one could then fabricate implants (Supplementary Fig. 3c).

### Fabrication of implants

Flexible implants were fabricated from a photocurable polymer (Ostemer 220, Mercene Labs AB, Sweden). Polymerization occurs when a mixture of two components (Part A and Part B) are exposed to UV light (Supplementary Fig. 4a-i). The PDMS template was silanized (trichloro(1H,1H,2H,2H-perfluorooctyl) silane, Sigma-Aldrich, Germany) for 1 h in a vacuum desiccator (Supplementary Fig. 4a-ii). Part A was warmed at 48 °C overnight to make sure there were no undissolved crystals remaining in the solution. Part B and the container were also heated up to 48 °C before mixing. Parts A and B were then mixed thoroughly and the mixture was degassed in a vacuum chamber for 5 min. A 200 μL drop of the mixture (1.86:1, wt\wt) was poured onto the template (Supplementary Fig. 4a-iv) and the template was mechanically sandwiched between two glass slides using two clips. The glass slide touching the implant polymer was previously plasma treated (PDC-32G, Harrick Plasma,USA) at 29 W for 1 min to facilitate implant release by improving the adhesion between the glass and implants. The solution was exposed to UV light (365 nm, UV9W-21, Lightning Enterprises, USA) for 10 min for polymerization (Supplementary Fig. 4a-v). The samples were rotated several times during UV exposure to ensure a homogeneous reaction throughout the template. The implants were released by mechanically agitating the templates in isopropyl alcohol (IPA) using a sonicator (DT 100 H, Bandelin Sonorex Digitec, Germany) (Supplementary Fig. 4a-vi). This whole process yielded a wafer with 100 implants (Supplementary Fig. 4b, c) that were subsequently cut out using a razor blade prior to implantation.

### Fabrication of a manipulator arm that temporarily displaces thoracic organs

We designed and constructed a manipulator arm to temporarily displace thoracic organs during implantation (Supplementary Fig. 5a, b). To construct the arm, we first 3D printed a mold that allowed us to glue a dissection pin (26002-10, Fine Science Tools, Germany) to the tip of a syringe needle (15391557, Fisher Scientific, USA) in a reproducible manner (Supplementary Fig. 5c). The pin is inserted into the needle until its tip touches the end of the mold. We glued the pin to the needle using a UV-curable adhesive (Bondic, Aurora, ON Canada). The arm was then bent using forceps and guided by a second 3D printed mold (Supplementary Fig. 5d). The pin was first bent coarsely and then adjusted more finely using the 3D printed mold. Another 3D printed piece was then used to connect the syringe needle to a 3-axis micromanipulator (DT12XYZ, ThorLabs, USA) and to an extension stage (Supplementary Fig. 5a). The whole structure was then attached to a breadboard (MB1224, ThorLabs, USA) (Supplementary Fig. 5b).

### Fabrication of a remounting stage

We used direct laser writing^65^ to fabricate a custom compliant mechanism that holds flies in place during two-photon microscopy. The mechanism was designed using 3D CAD software (SolidWorks 2021, Dassault Systémes, France). A 25 mm × 25 mm diced silicon wafer was used as the substrate upon which structures were printed. The surface of the substrate was plasma treated at 500 W for 7 min and coated with an aqueous solution of 10% (wt/vol) Poly(acrylic acid) (MW 50000, Polysciences, USA) at 2000 rpm for 15 s using a spin-coater (WS-650-23, Laurell Technologies Corporation, USA) (Supplementary Fig. 6a-i-iii). The mechanism was fabricated using a direct laser writer (Photonic Professional GT+, Nanoscribe GmbH, Germany) that controls two-photon polymerization (Supplementary Fig. 6a-iv). A polymer (IP-S, Nanoscribe GmbH, Germany) was chosen as the print material due to its Young’s modulus of 4.6 GPa^66^ and the resolution at which structures could be printed. The overall design was segmented into multiple frames because the maximum laser scan area provided by a 25× objective (NA 0.8, Zeiss) is 400 μm. This approach results in fine printing over a relatively large layout. The objective was dipped into liquid photoresist during printing. At the end of the printing process, the uncured polymer was removed using a developer (PGMEA, Sigma-Aldrich, Germany) for 20 min (Supplementary Fig. 6a-v). Finally, the PGMEA was rinsed using IPA. The mechanism was released from the substrate by dissolving the sacrificial layer in DI water (Supplementary Fig. 6a-vi). This yielded a microfabricated structure large enough to contain the thorax of the fly (Supplementary Fig. 6b, c). The remounting stage was completed by attaching the mechanism onto a laser-cut aluminum frame using UV-curable glue (Bondic, Aurora, ON Canada).

The materials and equipments required to fabricate our microengineered devices are summarized in Supplementary Tables 2 and 3.

### Implantation procedure

The steps required to prepare flies for long-term VNC imaging are described here (Fig. 1e, the internal organs have been sketched using data from ref. 67). See Supplementary Movie 2 for more details.

#### Tethering flies onto the dissection stage

A fly was cold anesthetized for 5 min. Then it was positioned onto the underside of a dissection stage and its wings were removed near their base using forceps. The thorax was then pressed through a hole (Etchit, Buffalo, MN) in the stage’s steel shim (McMaster-Carr, USA; 0.001” Stainless Steel, type 316 soft annealed; Part *#*2317K11). Afterwards, the stage was turned upside down and a tiny drop of UV-curable glue (Bondic, Aurora, ON Canada) was placed onto the scutellum, to fix the fly in place.

#### Opening the thoracic cuticle

The stage was filled with saline solution (Supplementary Table 1). A 30 G syringe needle was then used to cut a small rectangular hole (smaller than the 600 μm diameter window) into the dorsal thoracic cuticle. The hole was made by inserting the needle into the posterior thorax close to the scutellum. Then three lines were cut into the lateral and anterior thorax. A final line was cut to complete a rectangular opening. The resulting piece of cuticle was then removed using forceps.

#### Clearing out thoracic tissues

Residual degraded IFMs were removed from the opened thorax using forceps. Then, a pulled (P-1000, Sutter instrument, USA) glass needle (30-0018, Harvard Apparatus, USA) was used to detach small tracheal links between a large piece of trachea and the left side of the gut. The left salivary gland was then also removed using forceps.

#### Displacing thoracic organs using the manipulator arm

The manipulator arm was positioned on top of the stage with its tip visible. The dissection stage was positioned with the fly’s head pointing toward the experimenter. The arm tip was then inserted into the thorax using a 3-axis manipulator (DT12XYZ, ThorLabs, USA). The tip of the arm was then inserted to the (experimenter’s) right side of the gut near the middle of the proventriculus. The tip was inserted deep enough to be below the crop and salivary glands but not to touch the VNC. Once the tip of the arm was on the right side of the salivary gland, crop, and gut, it was pulled towards the left side of the thoracic cavity, making a space for the closed implant.

#### Positioning the implant

Once the flies’ organs were held securely onto the left side of the thoracic cavity by the manipulation arm, the implant was closed in the air using forceps and then transferred into the saline solution filling the dissection stage. The closed implant was then positioned in front of the fly on the stage. A thinner pair of forceps was next used to insert the implant into the animal’s thorax. Finally, a glass needle was used to adjust the location of the implant and to keep it at the appropriate height, allowing it to open passively. Once open, the glass needle was used to gently press the left side of the implant towards the bottom of the thorax while the arm was removed, and to remove any bubbles on the implant.

#### Sealing the thoracic hole with a numbered, transparent window

Once the implant was well positioned, a syringe needle (15391557, Fisher Scientific, USA) was used to remove saline solution from the stage. A window was then positioned on top of the cuticular hole and centered with the identification number on the posterior of the thorax near the scutellum. A wire was then used to add tiny drops of UV curable glue between the window and the surrounding thoracic cuticle, beginning from the right side of the scutellum and finishing on the left side. Saline solution was then added back to the stage. The cured UV glue, previously tethering the fly to the stage, was removed using a needle. The saline solution was then also removed and the window was fully sealed by placing and curing UV glue onto the fly’s posterior cuticle near the scutellum.

#### Dismounting flies from the dissection stage

Once the thoracic hole was fully sealed by a transparent window, the fly was dismounted from the dissection stage by gently pushing the front of the thorax through the hole in the steel shim. The fly was then returned to a vial of food to recover.

### *Drosophila melanogaster* experiments

All flies were raised on standard cornmeal food on a 12 h light:12 h dark cycle. Experiments for each particular study were performed at a consistent time of day to exclude the possibility of circadian-related confounding factors. No specific ethical approval was required for *Drosophila* experiments. For most experiments, female flies were used because they are larger in size. This increases the ease of dissection, implantation, and tethering. It also facilitates computational image processing and neural region-of-interest detection.

### Long-term study of survival and locomotion

Female flies expressing CsChrimson in Moonwalker Descending Neurons (MDNs)^28^ (*UAS-CsChrimson/Act88F-Rpr; VT50660.p65AD(attp40)/+; VT44845.Gal4DBD(attp2)/+*) (Fig. 2) were implanted at five days-post-eclosion (dpe). For this experiment, before implantation, implants were dipped in a 30 mg/ml dextran solution (*#*31392, Sigma-Aldrich, Switzerland) while mechanically closed. Implants were then taken out of the solution and dried using a twisted Kimwipe (5511, Kimberly-Clark, USA). This step was performed to fix implants in a closed position. However, we later discovered that dextran is not required to close implants and we removed this step. Implants were then positioned in the fly’s thorax as described above. The number of days following implantation is denoted as ‘days-post-implantation’ (dpi). Age and gender-matched control animals were selected from the same parental cross. For longevity studies, flies were housed individually in food vials and assessed every 1–2 days.

Studies of locomotion were performed at 1–3 dpi, 14–16 dpi, and 28–30 dpi. Animals were individually cold-anaesthetized and then transferred to rounded square arenas for optogenetic activation and video recording. Each recording consisted of 30 s of spontaneously generated behaviors (primarily walking and grooming), followed by three 3 s periods of optogenetic stimulation at 590 nm (6 mW/cm^2^) with 10 s interstimulus intervals. Therefore, each recording session was 59 s long.

To process video data, flies’ centroids were tracked using a customized version of Tracktor^68^. Their orientations were then extracted using a neural network (implemented in PyTorch^69^) that was trained on hand-labeled data. The network consisted of two convolutional layers followed by three fully connected layers. All layers, except for the final one, were followed by a ReLU activation function^70^. We also applied dropout after the first two fully connected layers with 0.2 probability^71^. To train the network, we hand annotated a total of 300 samples in three orientations (head up, head down, and sideways). The grayscale images were then cropped using Tracktor centroid locations and resized to 32 × 32 pixels. During training, we randomly applied affine transformations (20 degrees of rotation, 5 pixels of translation, and 0.2 scaling factor), horizontal, and vertical flip augmentations with a 0.5 probability. We used PyTorch’s torchvision package for all data augmentation. The network was trained with cross-entropy loss using 80% of the data. We used an Adam optimizer with a learning rate of 0.001, without weight decay and learning rate drop^72^. We trained for 1000 epochs and selected the weights with the best test error.

Translational velocities were computed by applying a second order Savitzky–Golay filter with a first-order derivative to centroid positions. The sign for the velocity values was set to negative for movements counter to the animal’s heading direction. Flies that collided with the arena’s walls for more than 0.3 s during the stimulation period were excluded from analysis. Flies that were dorsally flipped (i.e., walking on the sigma-coated ceiling) during the stimulation period were also excluded from analysis. The ‘Backward walking response slope’ metric was calculated as the acceleration from the beginning of each stimulation period to the minimum velocity (maximum backward speed) reached on that period. The ‘Backward walking distance traveled’ metric was computed as the left Riemann sum of the velocity curves during each stimulation period. We only considered frames where the velocity was negative. Finally, the ‘Maximum negative translational velocity’ is the minimum velocity value reached on each stimulation period.

For studies of male survival and behavior, animals expressing Reaper in their indirect flight muscles (*Act88F-Rpr; UAS-GFP; +/+*) were implanted at 1 day-post-eclosion (dpe). Age and gender-matched control (‘Intact’) animals were selected from the same parental cross. Flies were housed individually in food vials and flipped every day into a new food vial while assessing their longevity (Supplementary Fig. 10). Spontaneous behavior was recorded for three males per group. These males were individually cold-anaesthetized and then transferred to rounded square arenas for video recordings. Each recording consists of 60 s of spontaneously generated behaviors.

### Long-term pan-neuronal anatomical imaging in the VNC

Female flies expressing GFP throughout the nervous system (*Act88F-Rpr/+; GMR57C10-Gal4/UAS-GFP; +/+*) (Fig. 1h) were implanted at 4–6 dpe and kept individually in food vials. At 1–3 dpi, 14–16 dpi, and 28–30 dpi, flies were tethered onto a remounting stage and 25 imaging volumes of 100 μm depth (1 μm stepsize) were acquired using a two-photon microscope (Bergamo II microscope, ThorLabs, USA) and a 930 nm laser (MaiTai DeepSee, Newport Spectra-Physics, USA) with 20 mW of power at the sample location. We acquired 0.1 volumes-per-second (vps) using a Galvo-Resonance scanner^23^. The 25 images per depth were then registered to one another using the HyperStackReg module in Fiji^73^ and a rigid body transformation. These registered images were next projected along the time axis into one standard deviation image. The resulting volume was then depth color-coded using Fiji’s Temporal-Color macro.

For experiments in male flies, we imaged animals expressing GFP throughout the nervous system (*Act88F-Rpr; GMR57C10-Gal4/UAS-GFP; +/+*) (Supplementary Fig. 10). These animals were implanted at 1 dpe and kept individually in food vials. At 1 dpi, 5 dpi, and 10 dpi, flies were tethered and a volumetric recording of the VNC was performed using a two-photon microscope at 930 nm with 11 mW of laser power. Flies were anesthetized using carbon dioxide (1.3l/min) applied ventrally while recording imaging volumes. Volumes consisted of 512 × 512 pixel frames taken every 1 μm over a total depth of 100 μm. Z-projections were then generated with depth color-coding using Fiji’s ‘Temporal-Color’ macro.

### Long-term pan-neuronal functional recording in the VNC

Flies expressing GCaMP6f and tdTomato throughout the nervous system (*Act88F-Rpr/+; GMR57C10-Gal4/UAS-GCaMP6f; UAS-tdTomato/+*) were implanted at 5 dpe (female), or 1 dpe (male) and then mounted onto the two-photon imaging stage at 1, 5 and 10 dpi (Female, Supplementary Movie 4; male, Supplementary Movie 5). One horizontal imaging plane of the prothoracic neuromere was acquired using a two-photon microscope at 930 nm with 25 mW of power. Three horizontal z-plane images were acquired using a Galvo-Resonance scanner and averaged into one frame at an imaging rate of 10.7 fps. Behavior frames were acquired simultaneously (as in ref. 23) at a rate of 80 fps.

### Long-term sparse functional recording in the VNC

A female fly expressing GCaMP6f and tdTomato in DNa01 descending neurons^23,48^ (*Act88F-Rpr/+; GMR22C05-AD-spGal4/UAS-GCaMP6f; GMR56G08-DBD-spGal4 / UAS-tdTomato*) was implanted at 3 dpe. The same fly was then mounted onto the two-photon microscope stage at 1, 3 and 5 dpi. One horizontal imaging plane of the prothoracic neuromere was acquired using a two-photon microscope at 930 nm with 28 mW of laser power. Horizontal plane images were acquired using a Galvo-Galvo scanner at an imaging rate of 5.6 fps. Behavior frames were acquired simultaneously (as in ref. 23) at a rate of 80 fps. AxoID was used to detect the neurons as regions-of-interest (ROIs) in two-photon microscope images and extract their fluorescence values^50^. The semi-automated neural fluorescence event classifier described in ref. 50 was used to detect neural activity events from fluorescence traces. These were then correlated to spherical treadmill rotations.

### Modifying the implant to image the VNC T2 and T3 neuromeres

To illustrate how other implant designs could be used to gain optical access to posterior regions of the VNC (Supplementary Fig. 9), a female fly expressing GFP throughout the nervous system (*Act88F-Rpr/+; GMR57C10-Gal4/UAS-GCaMP6f; UAS-tdTomato/+*) was dissected at 3 dpe. A drop of UV glue was cured onto the tip of an implant. Implantation was also slightly modified: once inserted in the fly’s thorax, the implant was pushed posterior with the cured glue resting against the interior cuticle of the scutellum, fixing the implant into position. The fly was then closed using a transparent window. An imaging volume (576 × 576 pixels and 100 μm deep) of the VNC was recorded using a two-photon microscope with a Galvo-Galvo scanner at 930 nm with 22 mW of laser power.

### Long-term study of chordotonal organ degradation in the VNC following leg amputation

Female flies expressing GFP in their chordotonal organs (*Act88F-Rpr/+; iav-Gal4/UAS-GFP; +/+*) (Fig. 3) were implanted at 1 dpe. A z-stack of the VNC was recorded at 1 dpi, using a two-photon microscope at 930 nm with 55 mW of laser power. Flies were anesthetized with carbon dioxide (1.8l/min) supplied ventrally while recording z-stacks. Z-stacks consisted of 576 × 384 pixel frames taken every 1 μm over a total depth of 100 μm (i.e., 100 frames per volume). The front left leg was then removed at the thorax-coxa joint using dissection scissors (*#*15300-00, Fine Science Tools, Germany). A second z-stack was then immediately recorded. Flies were kept individually in food vials and imaged every day using the same recording parameters until 15 dpi. Fiji’s linear stack alignment with the SIFT registration plugin^74^ was then used to register all the projected z-stacks to the first z-stack. A custom Python script was then used to draw and extract the mean fluorescence of specific regions of interest. Mean fluorescence within these regions were measured for each day and normalized across animals by dividing them by the mean fluorescence on the first day.

Flies’ nervous systems were dissected and fixed with paraformaldehyde (441244, Sigma-Aldrich, USA) at 20 dpi. Samples were stained against nc82 with mouse anti-nc82 primary antibody diluted at 1:20 in PBST solution and then followed by goat anti-mouse Alexa 633 conjugated secondary antibody diluted at 1:500 (detailed procedure described in ref. 23). This allowed us to acquire confocal images that included both neuropil landmarks and endogenous GFP expression. Zen 2011 14.0 software was used to acquire confocal images. Confocal laser intensities and PMT gains were manually selected to avoid pixel saturation. These confocal z-stacks were then projected into 2D using Fiji’s standard deviation projection. The standard deviation projection of GFP expression is shown as an inverted image (Fig. 3c, d). A custom python script was written to detect the VNC’s boundaries using the standard deviation projection of nc82 images. This contour was detected using the Open CV library and then drawn onto GFP standard deviation projection images.

### Recording neural population activity before, during, and after feeding

Female flies (5 dpe) expressing a calcium indicator, GCaMP6f, and an anatomical marker, tdTomato, throughout the nervous system (*Act88F-Rpr/+; GMR57C10-Gal4/UAS-GCaMP6f; UAS-tdTomato/+*) (Fig. 4) were starved for 21–23 h on a wet Kimwipe (5511, Kimberly-Clark, USA). They were then implanted without a thoracic window, and kept on the dissection stage (the remounting stage was not used here) to limit the number of interventions. Animals were then positioned under a two-photon microscope where they could walk on a spherical treadmill consisting of an air-supported (0.8 L/min) foam ball (Last-A-Foam FR7106, General Plastics, USA) with a diameter of 1 cm^23^. Coronal cross-sections of their cervical connective were then imaged at 930 nm with a laser power of 15 mW. We achieved a 16 frames-per-second (fps) imaging rate by using a Galvo-Resonance scanner. In parallel, the behavior of the flies was recorded using seven cameras at 80 fps. Ball rotations were also measured along three axes using two optic flow sensors^6,23^. We recorded neural activity and behavior in trials of ~4 min each. First, four trials were recorded. Then, the foam ball was lowered and recording continued while flies fed on a solution consisting of either (i) 1 ml deionized water, 8 mg of sucrose (A2188.1000, Axon Lab, Switzerland) and 1 mg of Amaranth dye (A1016, Sigma-Aldrich, USA), (ii) a low concentration caffeine solution consisting of 1 ml deionized water, 8 mg caffeine (C0750, Sigma-Aldrich, USA), 8 mg of sucrose and 1mg of Amaranth, or (iii) a high concentration supersaturated caffeine solution consisting of 1 ml deionized water, 40 mg caffeine, 8 mg of sucrose and 1 mg of Amaranth. Animals were fed using a pulled glass needle (P-1000, Sutter instrument, USA; puller parameters- Heat: 502; Pull:30; Velocity: 120; Time: 200; Pressure: 200). A tiny drop of UV curable glue (Bondic, Aurora, ON Canada) was added near the tip of the needle to prevent the solution from traveling up on the needle. The needle was positioned in front of the flies using a manipulator (uMp-3, Sensapex, Finland). After feeding, the spherical treadmill was repositioned below the fly and eight more imaging trials were acquired.

#### Motion correction of two-photon imaging data

We used custom Python code unless otherwise indicated. For all image analysis, the y-axis is ventral-dorsal along the fly’s body, and the x-axis is medial-lateral. Image and filter kernel sizes are specified as (y, x) in units of pixels. Recordings from the thoracic cervical connective suffer from large inter-frame motion including large translations, as well as smaller, non-affine deformations. Because calcium indicators (e.g., GCaMP6f) are designed to have low baseline fluorescence, they are challenging to use for motion correction. Therefore, we relied on signals from the co-expressed red fluorescent protein, tdTomato, to register both the red (tdTomato) and the green (GCaMP6f) PMT channel images. First, we performed center-of-mass (COM) registration of each recorded frame to remove large translations and cropped the background regions around the neck connective (from 480 × 736 to 352 × 576). Then, we computed the motion field of each red frame relative to the first recorded frame using optic flow and corrected both red and green frames for the motion using bi-linear interpolation. The algorithm for optic flow motion correction was previously described in ref. 23. We only used the optic flow component to compute the motion fields and omitted the feature matching constraint. We regularized the gradient of the motion field to promote smoothness (*λ* = 800). Python code for the o ptic f low motion co rrection (ofco) package can be found at https://github.com/NeLy-EPFL/ofco.

#### Correction for uneven illumination

We observed that absolute fluorescence values were slightly lower on the right side of the connective than the left side, likely due to scattering by thoracic organs that are pushed to the right by the implant. To correct for this uneven absolute fluorescence, we computed the mean of all motion corrected frames across time. We then median filtered and low-pass filtered the resulting image (median filter: (71,91), Gaussian filter: *σ* = 3) to remove the features of individual neurons and retain only global, spatial changes in fluorescence. We then computed the mean across the y axis to obtain a fluorescence profile in the x (left - right) axis and fit a straight line to the most central 200 pixels. To correct for the decrease in fluorescence towards the right side, we multiplied the fluorescence with the inverse value of this straight line fit to the x-axis profile. Note that this correction only aids in the visualization of fluorescence, and does not have any impact on the computation of Δ*F*/*F* because, for a given pixel, both the fluorescence at each time point, and its baseline fluorescence are multiplied by the same constant factor.

#### Denoising calcium imaging data

To denoise registered and corrected data, we used an adapted version of the DeepInterpolation algorithm^75^. Briefly, DeepInterpolation uses a neuronal network to denoise a microscopy image by "interpolating" it from temporally adjacent frames. A U-Net is trained in an unsupervised manner using 30 frames (around 2 s) before and 30 frames after the target frame as an input and the current frame as an output. Thus, independent noise is removed from the image and components that dynamically evolve across time are retained. We modified the training procedure to fit one batch into the 11GB RAM of a Nvidia GTX 2080TI graphics card: rather than use the entire frame (352 × 576 pixels), we used a subset of the image (352 × 288 pixels) during training. We randomly selected the x coordinate of the subset. During inference, we used the entire image. We verified that using different images sizes during training and inference did not change the resulting denoised image outside of border regions. We trained one model for each fly using 2000 randomly selected frames from one of the trials before feeding and applied it to all of subsequent frames. Training parameters are outlined in Table 1. The adapted DeepInterpolation algorithm can be found on the "adapttoR57C10" branch of the following GitHub repository: https://github.com/NeLy-EPFL/deepinterpolation

**Table 1:** Training parameters for DeepInterpolation

Parameter	Value
Number of training frames	2000
Number of frames pre/post current frames	30
Omission of frames pre/post current frame	0
Number of iterations through training data	1
Learning rate	0.0001
Learning decay	0
Batch size	4
Steps per epoch	5
Number of GPUs	1
Number of workers	16

#### Generating Δ*F*/*F* videos

We show fluorescence values as Δ*F*/*F* (Supplementary Movies 10–12). This was computed as ${{\Delta }}F/F=\frac{F-{F}_{0}}{{F}_{0}}$, where *F* is the time varying fluorescence and *F*_0_ is the pixel-wise fluorescence baseline. To compute *F*_0_, we applied a spatial Gaussian filter (*σ* = 10) to images and convolved each pixel with a temporal window of 10 samples (around 0.6 s). We then identified the minimum fluorescence of each pixel across all trials.

#### Optic flow processing and classification of stationary periods

Optic flow sensors have been used to measure spherical treadmill rotations^6,23^ but they are inherently noisy. Therefore, we computed the moving average across 80 samples (around 200 ms). From preprocessed sensor values, we computed the forward, sideways and turning velocities^6^. We classified stationary periods (no movements of the ball) as periods when the absolute optic flow values of spherical treadmill rotation velocities were below a threshold of $0.31 {{{{{{{{\rm{ms}}}}}}}}}^{-1}\widehat{=}0.01$ rotations/s and at least 75% of the frames within the time ± 0.5 s of the sample were below this threshold. The latter criterion ensured that short stationary periods between bouts of walking would be excluded.

#### Synchronization of two-photon, optic flow, and camera data

We recorded three different data modalities at three different sampling frequencies: two-photon imaging data was recorded at ~16 Hz, behavioral images from seven cameras were acquired at 80 Hz, and ball movements using two optical flow sensors were measured at nearly 400 Hz. Therefore, to synchronize these measurements for further analysis, we down-sampled all measurements to the two-photon imaging frame rate by averaging all behavioral and ball rotation samples acquired during one two-photon frame.

#### Data analysis for caffeine ingestion experiment

To compute normalized fluorescence traces for each trial—as shown in Fig. 4e—we averaged the fluorescence across the entire cervical connective and computed the 99% percentile of this time series during trials before feeding. We then normalized the time series of all trials recorded from that fly to the 99% pre-feeding percentile. To perform statistical analysis, we used normalized fluorescence and computed the maximum (upper boundary of the 99% percentile) within certain time periods before, during, and after feeding. Before feeding, the maximum normalized fluorescence is unity for each of the 9 flies, as expected from the percentile normalization. For periods during, <9 min after, <19 min after, <29 min after, and <38 min after feeding, we performed Mann–Whitney *U* tests to determine whether the maximal neural activity after high caffeine ingestion was significantly different from the maximal activity after sucrose, or low caffeine ingestion (Fig. 4g). Aiming to apply the least amount or pre-processing necessary, until this point, we used fluorescence data that was not denoised using DeepInterpolation. However, in this case, to analyze the precise timing of the waves, we applied DeepInterpolation as described above. This reduces background fluorescence and high frequency noise. To analyze the temporal progression of fluorescence waves, we first identified the time of peak fluorescence across the entire cervical connective *T*_*peak*_. All times are given relative to the time of that peak permitting an analysis of precise timing differences. We then computed the mean fluorescence across time within manually selected regions of interest (dorsal, lateral, and ventral connective, as well as giant fiber neurons) and represent them normalized to their minimum and maximum values. We smoothed the time series with a Gaussian filter ($\sigma=3\widehat{=}0.18$ s). To identify the peak time for each pixel, we applied a temporal Gaussian filter ($\sigma=10\widehat{=}0.62$ s) and spatial Gaussian filter (*σ* = 1) and searched for the maximum fluorescence value within *T*_*peak*_ ± 10 s. In Fig. 4f we show the mean fluorescence during periods when the fly was stationary (i.e., not moving the ball).

### Reporting summary

Further information on research design is available in the Nature Research Reporting Summary linked to this article.

## Supplementary information

Supplementary Information

Description of Additional Supplementary Files

Supplementary Movie 1

Supplementary Movie 2

Supplementary Movie 3

Supplementary Movie 4

Supplementary Movie 5

Supplementary Movie 6

Supplementary Movie 7

Supplementary Movie 8

Supplementary Movie 9

Supplementary Movie 10

Supplementary Movie 11

Supplementary Movie 12

Supplementary Movie 13

Reporting Summary

## Data Availability

The raw data generated in this study have been deposited on a public repository available at: https://dataverse.harvard.edu/dataverse/long_term_imaging_vnc_drosophila. Source data are provided with this paper.

## Source data

Source Data

## Code availability

Custom made codes used to analyze the raw data are available at: https://github.com/NeLy-EPFL/Long-Term-Imaging-VNC-Drosophila10.5281/zenodo.6826488.
