# Supplementary material for: Microengineered devices enable long-term imaging of the ventral nerve cord in behaving adult Drosophila
Source: Nat Commun. 2022 Aug 25;13:5006. doi: 10.1038/s41467-022-32571-y (PMC9411199; doi:10.1038/s41467-022-32571-y)
Supplement: Supplementary file 1 — Supplementary Information [file 41467_2022_32571_MOESM1_ESM.pdf]

Supplementary information

Microengineered devices enable long-term imaging of the ventral nerve cord in behaving adult *Drosophila*

Laura Hermans *[1,2], Murat Kaynak *[2], Jonas Braun[1], Victor Lobato Ríos[1], Chin-Lin Chen[1], Adam Friedberg[1], Semih Günel[1,3], Florian Aymanns[1], Mahmut Selman Sakar †[2], and Pavan Ramdya †[1]

[1]Neuroengineering Laboratory, Brain Mind Institute & Interfaculty Institute of Bioengineering, EPFL, Lausanne, Switzerland
[2]Microbiorobotic Systems Laboratory, Institute of Mechanical Engineering & Interfaculty Institute of Bioengineering, EPFL, Lausanne, Switzerland
[3]Computer Vision Laboratory, EPFL, Lausanne, Switzerland

* equal contribution
† corresponding authors: pavan.ramdya@epfl.ch; selman.sakar@epfl.ch

Contents

1 Supplementary Tables **2**

2 Supplementary Figures **4**

1 Supplementary Tables

Supplementary Table 1: Saline solution

Chemical	mM
NaCl	103
KCl	3
NaHCO3	26
NaH2PO4	1
CaCl2 (1M)	4
MgCl2 (1M)	4
Trehalose	10
TES	5
Glucose	10
Sucrose	2

Supplementary Table 2: Main materials for long-term imaging tool fabrication

Device	Material	Part number	Company
Implant	Silicon Wafer	100/P/SS/01-100	Sigert Wafer, Germany
	HMDS	999-97-3	Sigma Aldrich, Germany
	Positive resist	AZ9260	Microchemicals GmbH, Germany
	Remover	Remover1165	Kayaku Advanced Materials, USA
	Silane	448931	Sigma Aldrich, Germany
	PDMS	01673921	Dow Europe GmbH, Germany
	Polymer	Ostemer 220	Mercene Labs AB, Sweden
Window	Silicon Wafer	100/P/SS/01-100	Sigert Wafer, Germany
	Dextran	205195	MP Biomedicals, USA
	Negative resist	SU8-3025	Kayaku Advanced Materials, USA
	Developer	PGMEA	Sigma Aldrich, Germany
	Positive resist	AZ40XT	Microchemicals GmbH, Germany
	Remover	Remover1165	Kayaku Advanced Materials, USA
Remounting stage	Silicon Wafer	100/P/SS/01-100	Sigert Wafer, Germany
	Poly(Acrylic acid)	9003-01-4	Polysciences, USA
	Polymer	IP-S	Nanoscribe GmbH, Germany
	Developer	PGMEA	Sigma Aldrich, Germany
	Glue	Bondic glue	Bondic, Aurora, Canada

Supplementary Table 3: Main equipment for long-term imaging tool fabrication

**Device**	**Equipment**	**Part number**	**Company**
Implant	Resist processing system	EVG 150	EV Group, Germany
	Etcher	AMS 200 SE	Alcatel, France
	Vacuum Pump	EV-A01-7	Swiss Vacuum Tech. SA, Switzerland
	Vacuum desiccator	F42020-0000, SP	Bel-Art, USA
	Oven	UF30	Memmert GmbH, Germany
	Plasma Cleaner	PDC-32G	Harrick Plasma, USA
	UV Light	UV9W-21	Lightning Enterprise, USA
	Sonicator	DT 100 H	Bandelin Sonorex Digitec, Germany
Window	Mask aligner	MJB4	Süss MicroTec, Germany
	Direct laser writer	VPG-200	Heidelberg Instruments, Germany
	Automatic mask processor	HMR900	HamaTech, Germany
	Optical microscope	DM8000 M	Leica Microsystems, Switzerland
	Mechanical surface profiler	Dektak XT	Bruker Corporation, USA
	Plasma stripper	PVA TePla 300	PVA AG, Germany
	Spin coater	WS-650-23	Laurell Technologies Corporation, USA
	Automated processing system	ACS200 Gen3	Süss MicroTec, Germany
	Vacuum Evaporation Machine	EVA 760	Alliance-Concept, France
Remounting stage	CAD Software	SolidWorks 2021	Dassault Systèmes, France
	Plasma stripper	PVA TePla 300	PVA AG, Germany
	Spin coater	WS-650-23	Laurell Technologies Corporation, USA
	3D writer	Photonic Professional GT+	Nanoscribe GmbH, Germany

2 Supplementary Figures

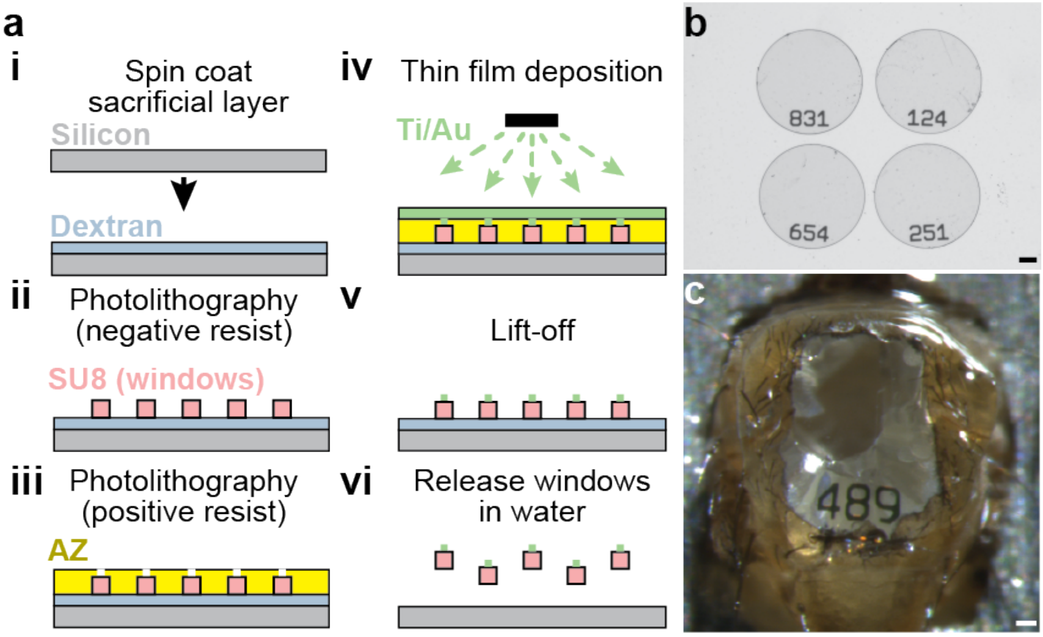

Supplementary Figure 1: **Fabrication of numbered, optically transparent thoracic windows.** **(a)** Thoracic windows are fabricated by performing the following steps. **(i)** A sacrificial layer of dextran is spin-coated onto a silicon wafer. **(ii)** SU-8 windows are structured onto the sacrificial layer, using photolithography. **(iii)** A positive resist, AZ, is cross-linked to mark number openings. **(iv)** Ti/Au is vapor deposited. **(v)** The AZ layer is lifted off. **(vi)** Finally, the numbered windows are released in water. **(b)** This process yields transparent SU-8 windows with thin Ti/Au numbers. Scale bar is 100 µm. **(c)** A window on an implanted animal, permitting a view of thoracic organs and tracking of this animal's identity. Scale bar is 50 µm.

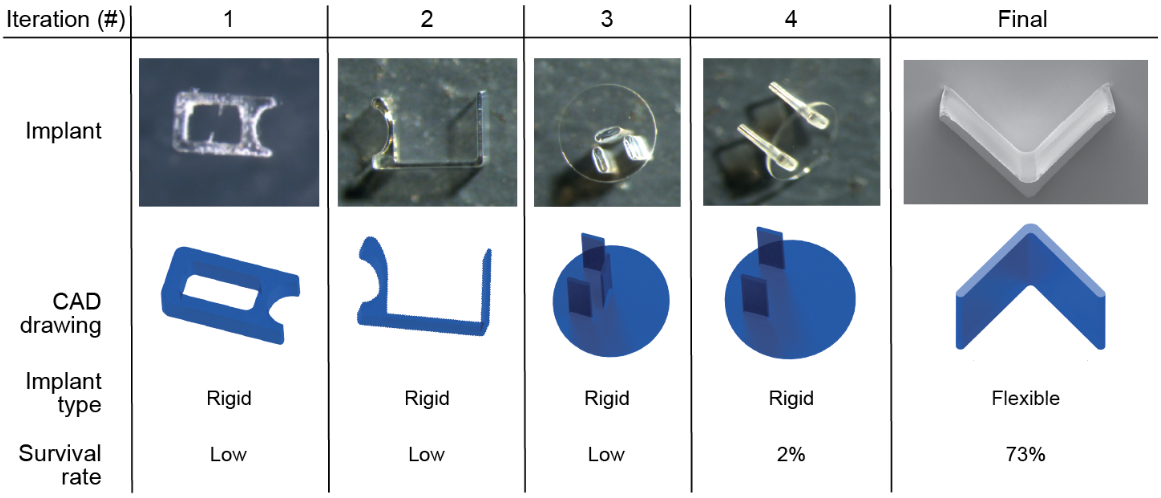

Supplementary Figure 2: **Design iterations of implants used to displace thoracic tissues and enable long-term VNC imaging.** Early designs were rigid (SU-8 based), with an open shape used to protect imaging regions of interest from invading tissues (iterations 1 and 2). Later rigid designs combined the thoracic window with protective pillars (iterations 3 and 4). All of these early iterations yielded single-digit survival rates. A major breakthrough was in the fabrication of compliant (Polymer-based) V-shaped implants. Through iterative testing of V-shaped implant sizes and angles that displace but do not squeeze internal organs, we converged upon the reported solution (iteration 'Final') with a high survival rate.

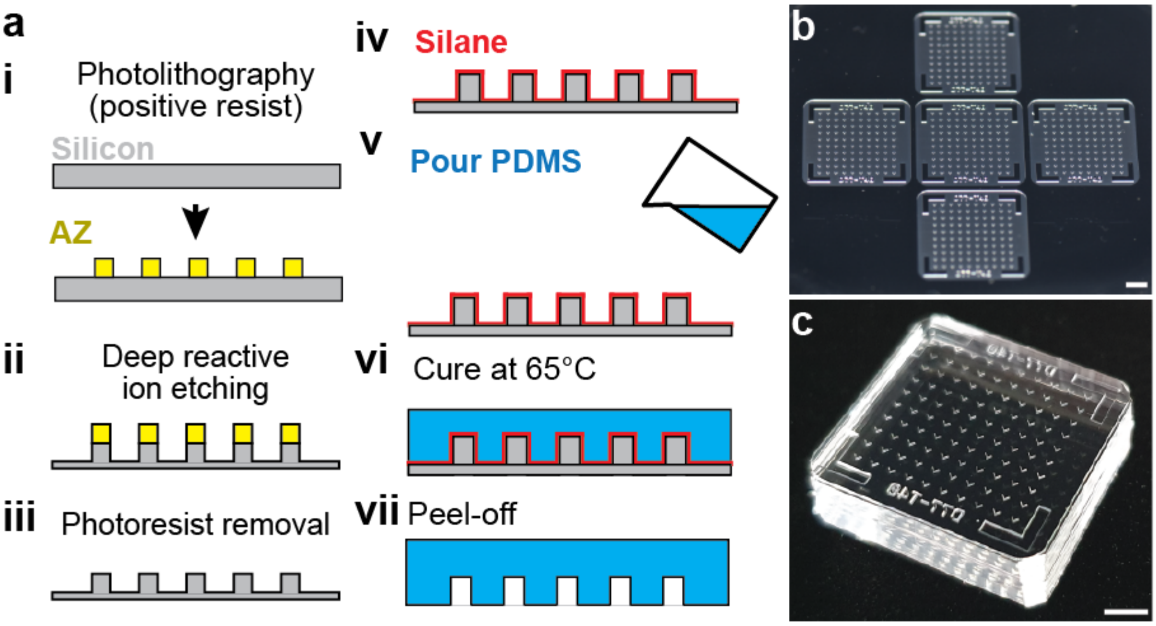

Supplementary Figure 3: **Fabrication of molds used to cast implants.** **(a)** Implant molds are fabricated by performing the following steps. **(i)** Through photolithography, a positive resist, AZ, is cross-linked onto a silicon wafer to form a temporary mask. **(ii)** Deep reactive ion etching is used to sculpt the silicon wafer. **(iii)** The photoresist is removed. **(iv)** Subsequently, this silicon piece is silanized. **(v)** PDMS is then poured, **(vi)** cured, and **(vii)** peeled off. **(b)** This process yields a single large piece. Scale bar is 0.5 cm. **(c)** This large piece is cut into five individual implant molds. Scale bar is 0.5 cm.

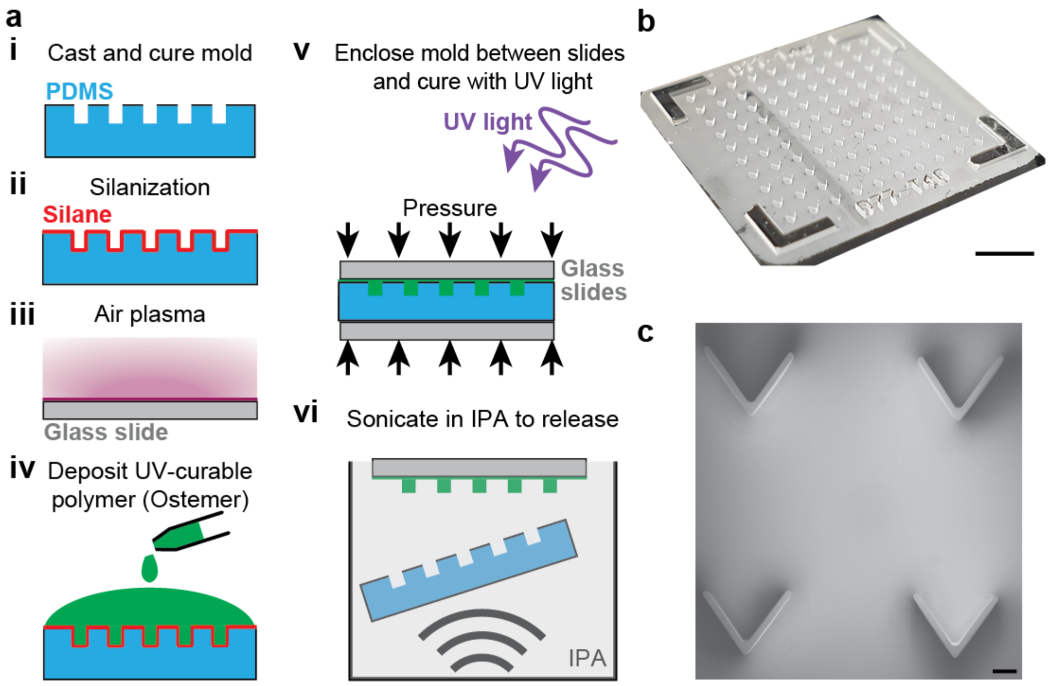

Supplementary Figure 4: **Fabrication of implants.** **(a)** Implants are fabricated by performing the following steps. **(i)** PDMS molds are cast, cured, and cut into pieces. **(ii)** PDMS molds are silanized. **(iii)** A glass slide is plasma treated to promote adhesion. **(iv)** A UV curable polymer is deposited onto the PDMS mold. **(v)** This composite is sandwiched between glass slides and exposed to UV light. **(vi)** The mold is sonicated to release in IPA. **(b)** This high-throughput process yields 100 implants in a single mold. Scale bar is 0.5 cm. **(c)** A scanning electron microscopy image confirms the precision of implant fabrication. Scale bar is 200 µm.

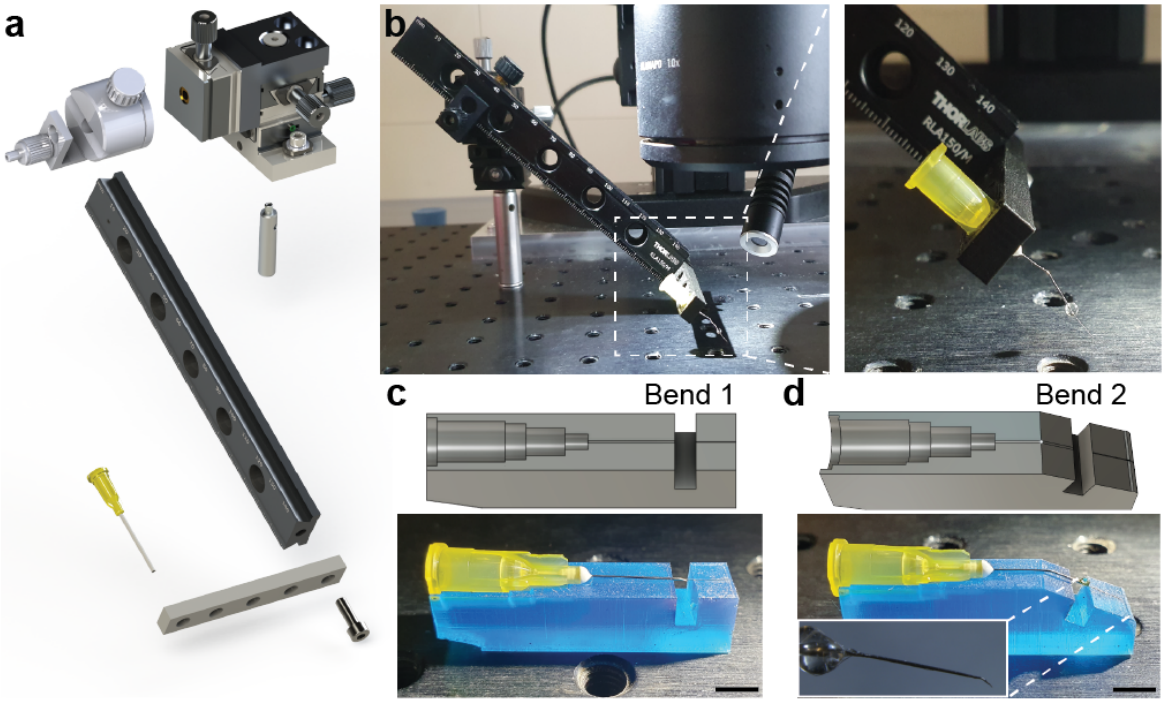

Supplementary Figure 5: **Fabrication of a manipulator arm to temporarily displace thoracic organs.** **(a)** Exploded view of the manipulator arm and its component parts. **(b)** (**left**) View of the manipulator arm mounted near the dissection microscope. (**right**) Zoomed in view of the inset (dashed white lines) highlighting the bent needle tip. **(c)** 3D printed piece used to guide gluing of the pin to the syringe needle. Scale bar is 0.5 cm. **(d)** 3D printed piece used to guide bending the manipulator arm tip. Inset shows a zoomed-in view of the arm's tip.

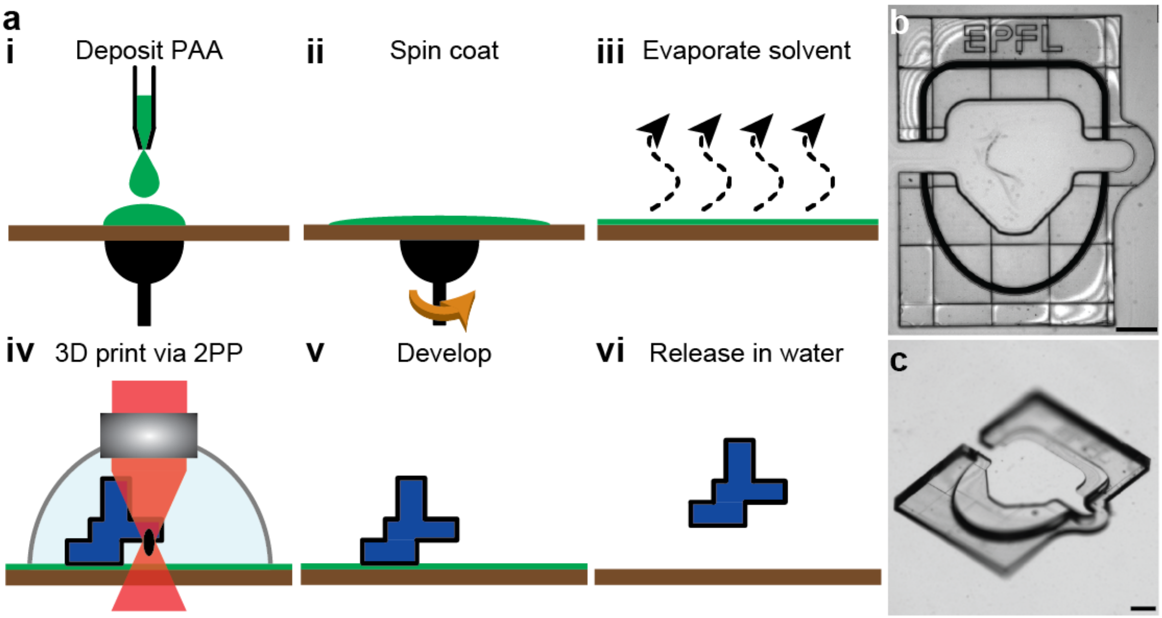

Supplementary Figure 6: **Fabrication of a remounting stage.** **(a)** A water soluble sacrificial solution is **(i)** deposited and **(ii)** spin-coated to ensure a thin layer. **(iii)** The water in the solution is evaporated, leaving a dry water soluble layer. **(iv)** The remounting stage is 3D printed using two-photon polymerization (2PP). **(v)** This is followed by development in a solvent. **(vi)** Finally, the piece is released in water. **(b)** A microscope image of the remounting stage before releasing it in water. Scale bar is 0.25 mm. **(c)** Another view of the remounting stage illustrating its ergonomic design for fly tethering. Scale bar is 0.25 mm.

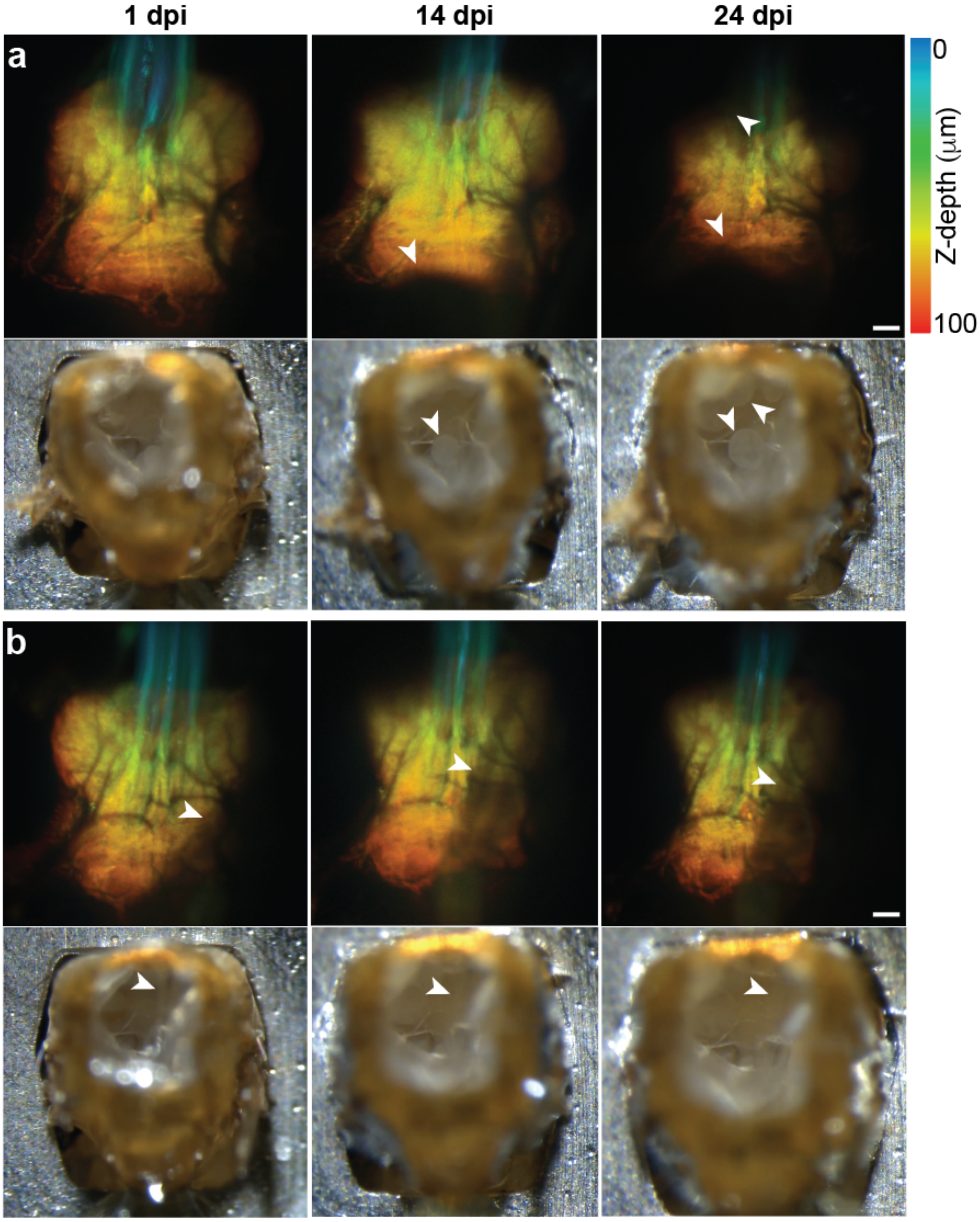

Supplementary Figure 7: **Potential organ movements within the thorax after implantation.** Two implanted animals at (**left**) 1 dpi, (**middle**) 14 dpi, and (**right**) 24 dpi. Highlighted are image-obscuring movements of the **(a)** fat bodies, or **(b)** salivary glands. **(top row)** Two-photon images of the animal's VNC expressing GFP throughout the nervous system. White arrowheads indicate **(a)** fat bodies, or **(b)** salivary glands that shift over time obscuring the view of the VNC. Z-stacks are depth color-coded (100 µm). Scale bar is 25 µm. (**bottom row**) The same animal's dorsal thorax, visualized using a dissection microscope.

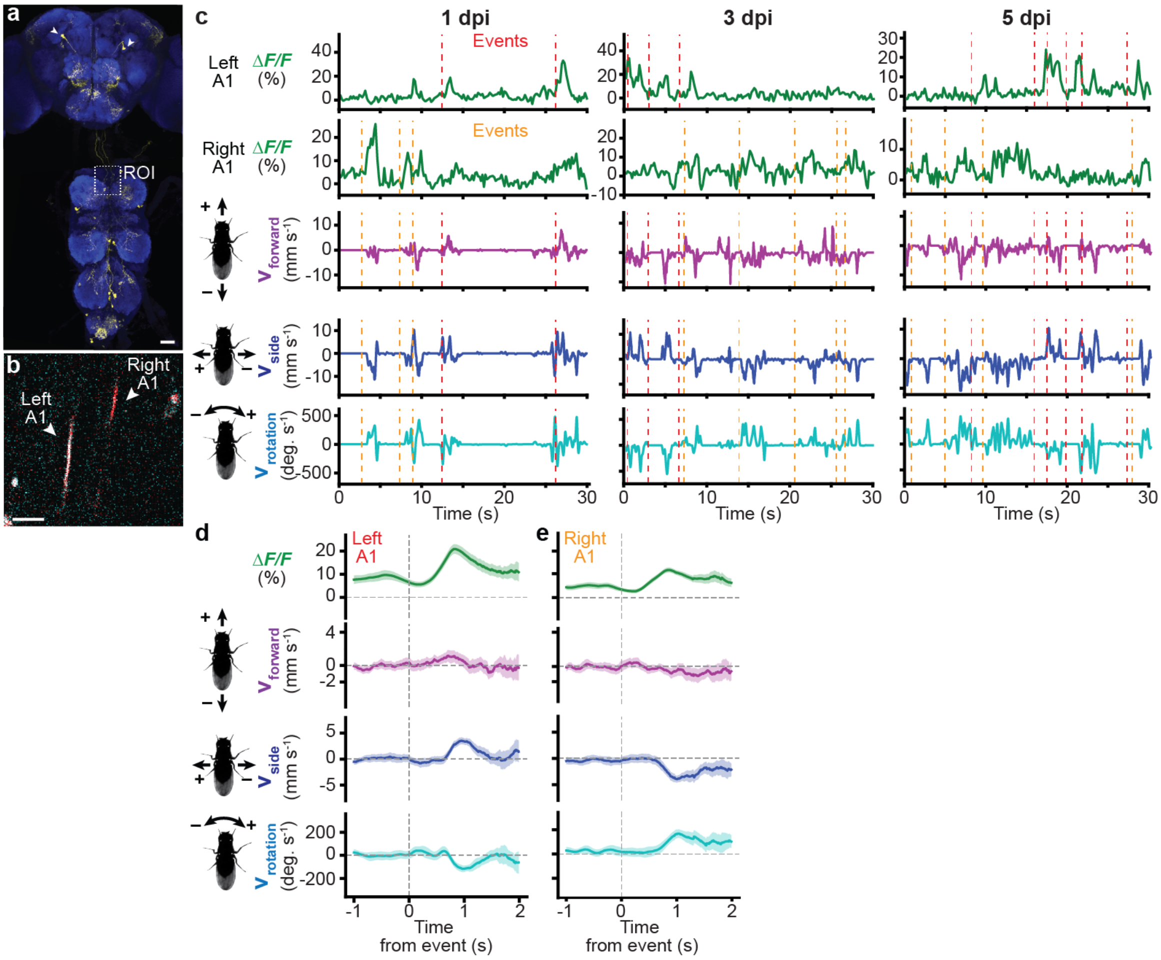

Supplementary Figure 8: **Repeated neural recordings of a pair of DNa01 descending neurons. (a)** Confocal image of DNa01-Gal4 driver line expression in the brain and VNC. Scale bar is 40 μm. Shown are neuronal GFP (yellow) and neuropil (nc82, blue). A dashed white box highlights the VNC imaging region of interest ('ROI'). Image is modified from [1]. **(b)** One two-photon horizontal image of the cervical connective. Indicated are the left and right DNa01 neuron axons (white arrow heads). Scale bar is 16 μm. **(c)** Activities of the left (**first row**) and right (**second row**) DNa01 neuron are synchronized with the fly's (**third row**) forward, (**fourth row**) sideways, and rotational (**fifth row**) velocities as measured by spherical treadmill ball rotations. Neural activity 'events' are indicated for left (red) and right (yellow) DNa01 neurons. Traces for one fly are shown for recordings at 1 (**left**), 3 (**middle**) and 5 (**right**) dpi. **(d,e)** All DNa01 **(d)** left and **(e)** right neuron activity events aligned at 0s. Solid lines and shaded regions show the mean and 95% confidence interval, respectively.

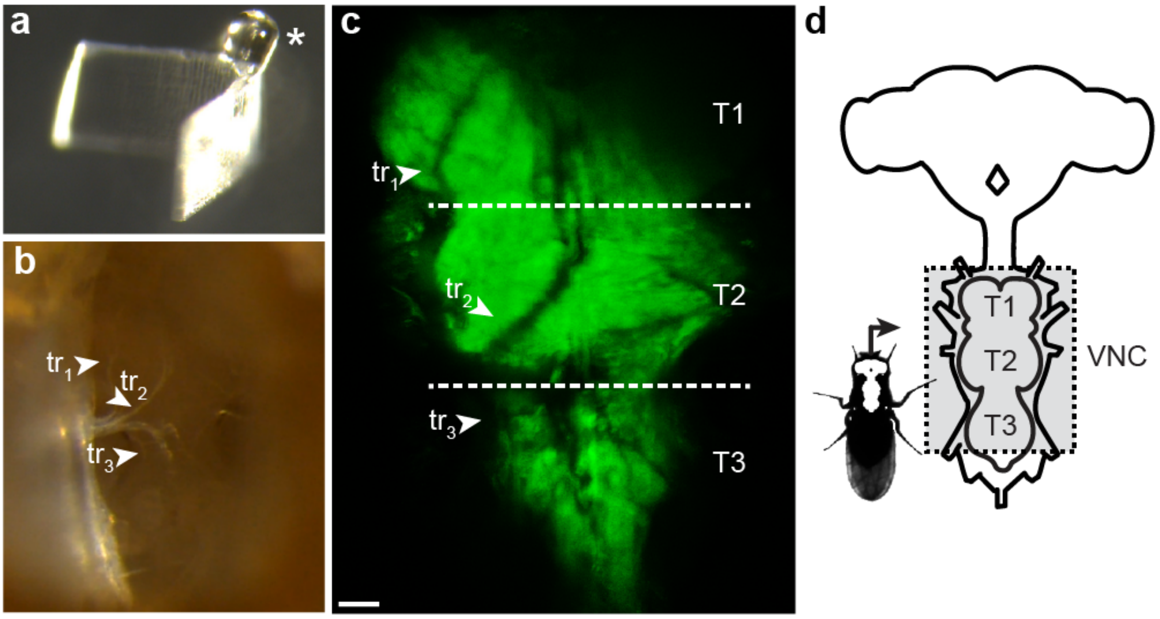

Supplementary Figure 9: **Recording posterior regions of the VNC.** **(a)** Prototype modified implant, with a drop of UV glue cured onto the apex of an implant. **(b)** Dissection microscope view through the dorsal thoracic window, revealing posterior positioning of the implant. Highlighted are tracheal fibers innervating the VNC (white arrowheads)(n=1). **(c)** Z-projected two-photon microscopy imaging volume of the VNC for the animal expressing GFP throughout the nervous system in panel B. Indicated are the same tracheal innervations from panel B (white arrowheads) as well as the newly visible T2 and T3 VNC neuromeres. Scale bar is 30 μm. **(d)** A schematic of the VNC region imaged in panel C.

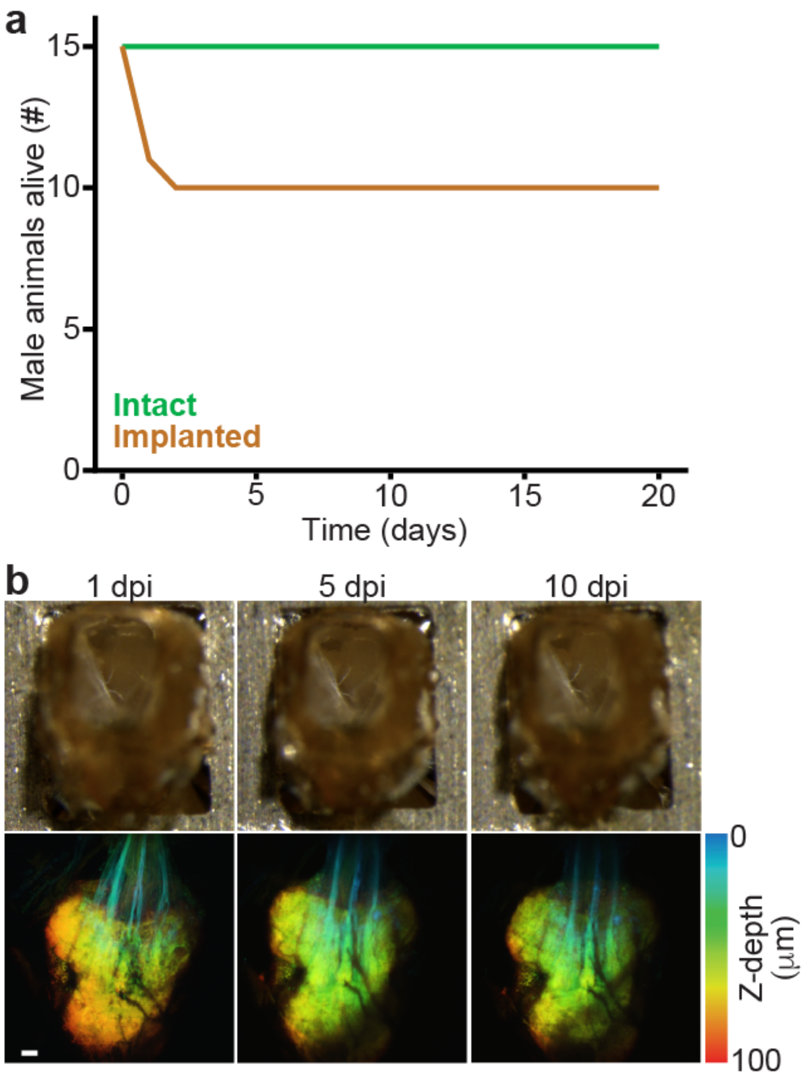

Supplementary Figure 10: **Implanted male fly survival and long-term anatomical imaging.** **(a)** Survival curves for genetically-identical sibling male flies that were either (i) not experimentally manipulated (green, 'Intact'), or (ii) prepared for long-term imaging by implantation and the addition of a thoracic window (orange, 'Implanted'). Source data are provided as a Source Data file. **(b)** The dorsal thorax of an implanted animal, as seen from the dissection microscope (**top row**), and the corresponding z-projected volumetric image of the VNC, visualized using a two-photon microscope (**bottom row**). This animal expressed GFP throughout the nervous system. Recordings were performed at (**left**) 1, (**middle**) 5, and (**right**) 10 dpi. Z-stacks are depth color-coded (100 μm). Scale bar is 20 μm.

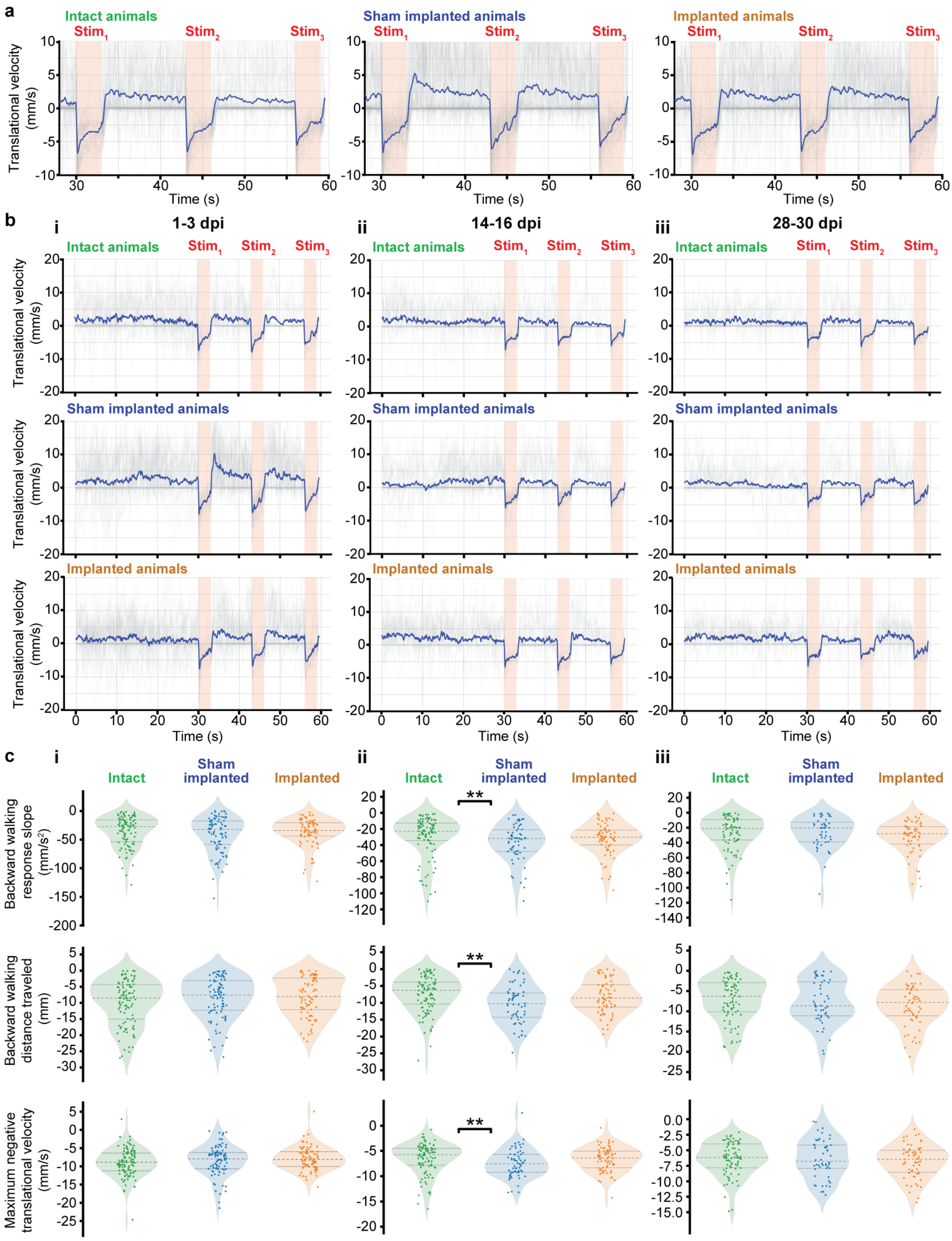

Figure S11 - See Figure Legend on next page.

Supplementary Figure 11: **Impact of implantation and windows on behavior, separated by age post-implantation.** **(a)** Zoomed in traces of translational velocities shown in Figure 2 for (**left**) intact, (**middle**) sham implanted, and (**right**) implanted flies including three recordings taken over one month. **(b)** Translational velocities of intact (**top**), sham implanted (**middle**), and implanted (**bottom**) animals during 30 s of spontaneous behavior, followed by three optogenetic stimulation periods of 3 s each (pink, 'Stim'). Shown are the raw (grey) and mean (blue) traces arranged by age: **(i)** 1-3 dpi, **(ii)** 14-16 dpi, or **(iii)** 28-30 dpi. **(c)** From these time-series data, we used each stimulation event as one data point for calculating summary statistics (1-3 dpi - intact group, n=103; sham implanted group, n=90, implanted group, n=81. 14-16 dpi - intact group, n=101, sham implanted group: n=66, implanted group: n=71; 28-30 dpi - intact group, n=82, sham implanted group, n=52, implanted group, n=61). Summary statistics include (**top**) the initial negative slope in translational velocity—backward walking—upon optogenetic stimulation, (**middle**) the integrated translational velocity over the entire optogenetic stimulation period, and (**bottom**) the peak negative translational velocity over the entire optogenetic stimulation period. Data are sorted by age as in panel B. A Kruskal-Wallis statistical test was used to compare behaviors across the three groups. A post-hoc Conover's test with a Holm correction was used to perform pairwise comparisons. Significant differences were found at 14-16 dpi between the two control groups: 'Sham implanted' and 'Intact'. One asterisk (*) indicates $P < 0.05$ and two asterisks (**) indicate $P < 0.01$. Source data are provided as a Source Data file.

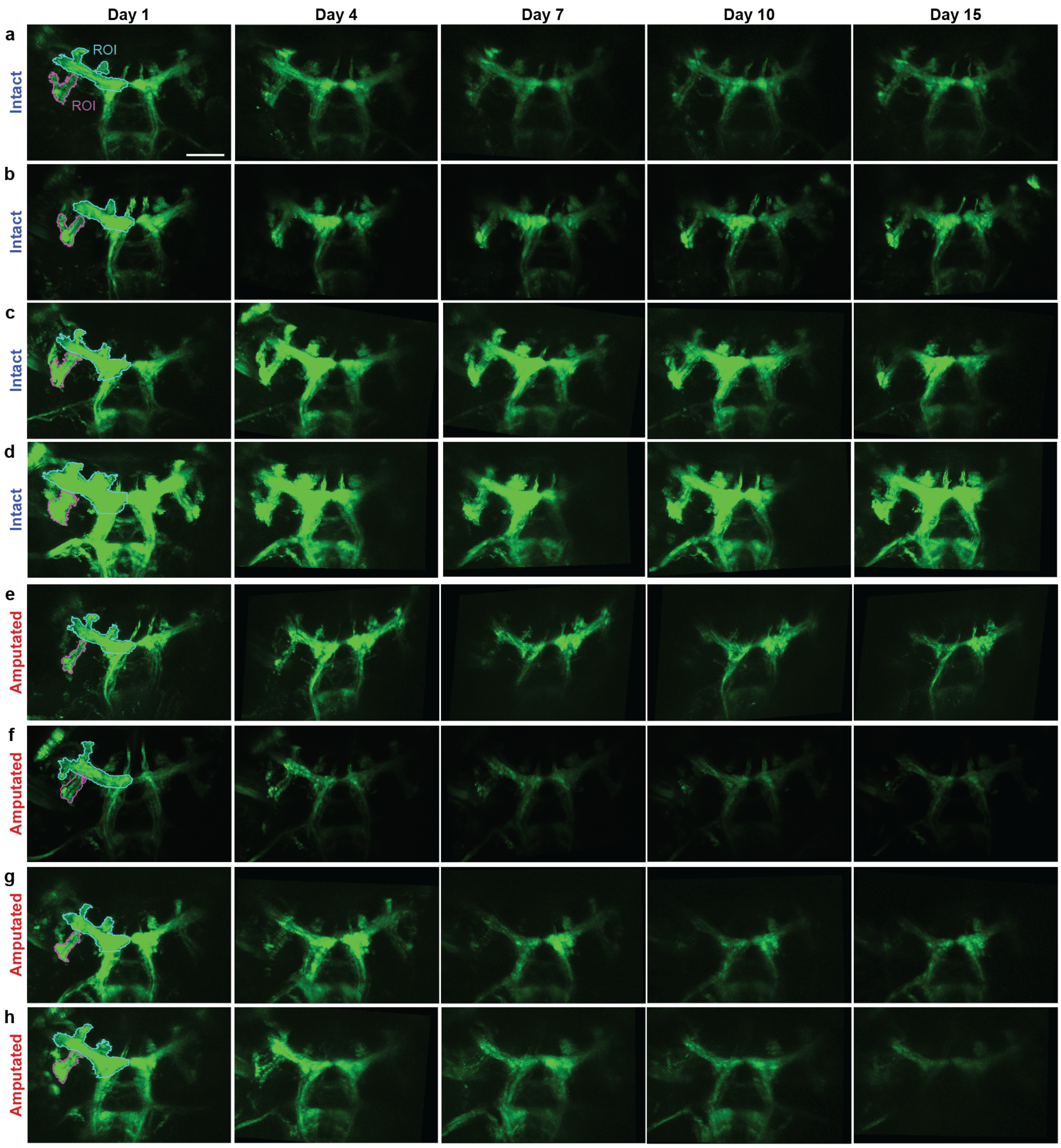

Supplementary Figure 12: **Long-term imaging of front leg chordotonal organs axon terminals in the VNC in intact or amputee animals.** Maximum intensity projections of z-stacks taken at 1, 4, 7, 10 and 15 dpi. Data are registered to images at 1 dpi. Scale bar is 50µm. Cyan and pink ROIs used for quantification in Figure 3 are indicated. Data are for **(a-d)** four control animals with intact legs and **(e-h)** four animals whose front left legs were amputated at 2 dpi.

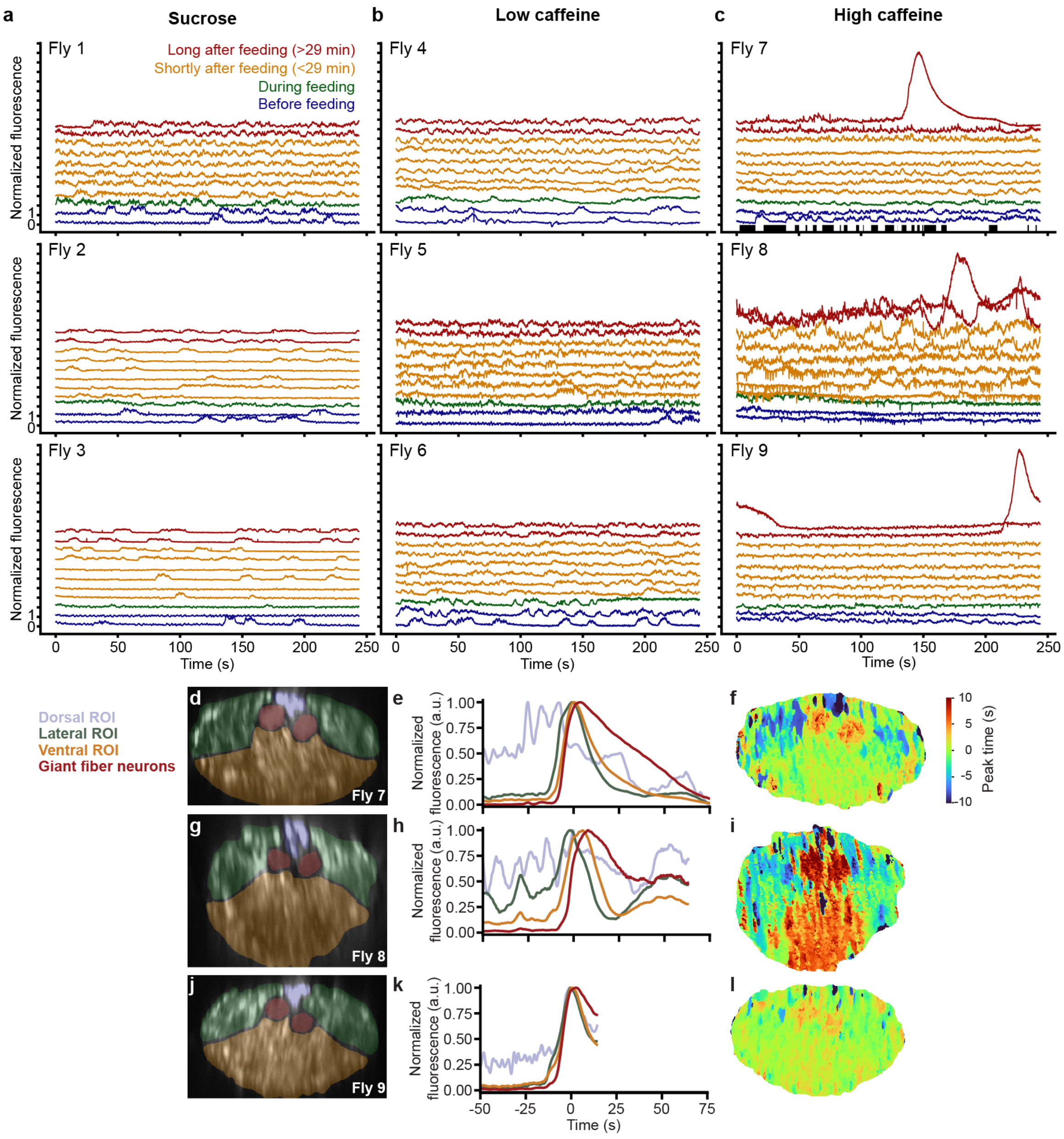

Supplementary Figure 13: **Waves of neural activity observed across three animals following ingestion of high-concentration caffeine.** **(a-c)** Normalized fluorescence across all axons passing through the thoracic neck connective over four minute recordings either before (blue), during (green), shortly after (orange), or long after (red) feeding. Three flies per condition were fed a solution containing either only sucrose **(a)**, or sucrose and **(b)** a low-dose, or **(c)** high-dose of caffeine. Flies 1, 4, and 7 are shown in Figure 4e. Black bars below Fly 7 traces show times when the fly was stationary during first trial. The Pearson correlation coefficient between the normalized fluorescence and time-series of stationary periods is $r = -0.67$, indicating that spontaneous fluctuations are strongly linked to changes in behavioral state. **(d,g,j)** Thoracic cervical connectives from three animals. ROIs overlaid on top of standard-deviation time-projected images. **(e,h,k)** Neural activity over time for each ROI (color-coded) normalized to the peak fluorescence during the wave of activity. Shown are three waves from three animals. Time is aligned to the peak of the mean fluorescence across all ROIs. **(f,i,l)** Pixel-wise time of peak activity (color-coded) relative to the peak of mean activity across the entire neck connective.

Supplementary References

[1] Chen, C.-L. *et al.* Imaging neural activity in the ventral nerve cord of behaving adult *Drosophila*. *Nature communications* **9**, 1–10 (2018).
