# Supplementary material for: Microengineered devices enable long-term imaging of the ventral nerve cord in behaving adult Drosophila
Source: Nat Commun. 2022 Aug 25;13:5006. doi: 10.1038/s41467-022-32571-y (PMC9411199; doi:10.1038/s41467-022-32571-y)
Supplement: Supplementary file 2 — Description of Additional Supplementary Files [file 41467_2022_32571_MOESM2_ESM.pdf]

1 Supplementary Movies

Supplementary Movie 1: Interactions among implanted and intact freely behaving animals. Two implanted animals—identifiable by visible thoracic windows—and one intact animal interact near a morsel of food. Video is real-time.

Supplementary Movie 2: Protocol to prepare animals for long-term neural recordings. A step-by-step visualization of how a fly is outfitted with an implant and window for long-term two-photon microscope recordings.

Supplementary Movie 3: Repeatedly recording VNC anatomy across one month. Two-photon z-stacks of an animal's VNC at 1, 14, and 28 days post-implantation (dpi). This animal expressed GFP throughout the nervous system (*GMR57C10-Gal4*). Z-stack images progress from the dorsal to ventral VNC.

Supplementary Movie 4: Repeatedly recording VNC neural activity in female flies across ten days. Two-photon imaging of a female animal's VNC at 1, 5, and 10 days post-implantation (dpi). This animal expressed a genetically-encoded calcium indicator, GCaMP6f, and an anatomical fiduciary, tdTomato, throughout the nervous system (*Act88F:Rpr; GMR57C10-Gal4 / UAS-GCaMP6f; UAS-tdTomato*). Neural data are averaged across three cumulatively acquired two-photon microscope images. Activity are related to foreleg-dependent grooming.

Supplementary Movie 5: Repeatedly recording VNC neural activity in male flies across ten days. Two-photon imaging of a male animal's VNC at 1, 5, and 10 days post-implantation (dpi). This animal expressed a genetically-encoded calcium indicator, GCaMP6f, and an anatomical fiduciary, tdTomato, throughout the nervous system (*Act88F:Rpr; GMR57C10-Gal4 / UAS-GCaMP6f; UAS-tdTomato*). Neural data are averaged across three cumulatively acquired two-photon microscope images. Activity are related to forward walking.

Supplementary Movie 6: Long-term imaging of DNa01 descending neuron axons. Two-photon horizontal imaging of DNa01 neuron axons within an animal's (*Act88F-Rpr/+; GMR22C05-AD-spGal4 / UAS-GCaMP6f; GMR56G08-DBD-spGal4 / UAS-tdTomato*) thoracic cervical connective at 1, 3, and 5 days post-implantation (dpi).

Supplementary Movie 7: Optogenetically elicited backward walking of intact, sham implanted, and implanted female flies. Representative videos of three female flies driven to walk backward through optogenetic activation of Moonwalker Descending Neurons. Columns are experimental dates (1, 14, and 28 dpi). Rows are experimental groups (Intact, Sham implanted, and Implanted). A light appears on the bottom-left of each arena, indicating times of orange light illumination and CsChrimson activation. Trajectories are shown for forward walking (cyan) and backward walking (purple).

Supplementary Movie 8: Spontaneous behaviors of intact and implanted male flies. Representative videos of two male flies behaving spontaneously. Columns are experimental dates (1, 5, 10, and 20 dpi). Rows are experimental groups (Intact and Implanted). Trajectories are shown for forward walking (cyan) and backward walking (purple).

Supplementary Movie 9: Repeatedly recording the anatomy of proprioceptive inputs to the VNC for 15 days before and after forelimb amputation. Two-photon z-stacks of two animals' VNCs at 1, 7, and 15 days-post-implantation (dpi). These animals expressed GFP in limb chordotonal organs (*iav-Gal4*). Z-stack images progress from the dorsal to ventral VNC. Top row shows data from an animal with an intact leg. Bottom row shows an animal whose front left leg was amputated at 2dpi.

Supplementary Movie 10: Repeatedly recording thoracic cervical connective neural activity before, during, right after, and long after feeding with a sucrose solution. Two-photon imaging of a cross-section of the thoracic cervical connective including neurons descending from and ascending to the brain. Columns are data acquired before (left), during (middle-left), right after (middle-right), and 25 minutes (right) after feeding with a sucrose solution. Rows are behavioral videography (top), $\Delta F/F$ (middle) and motion-corrected raw (bottom) two-photon calcium imaging data. This animal expressed GCaMP6s and tdTomato, throughout the nervous system.

Supplementary Movie 11: Repeatedly recording thoracic cervical connective neural activity before, during, right after, and long after feeding with a low-concentration caffeine and sucrose solution. Two-photon imaging of a cross-section of the thoracic cervical connective including neurons descending from and ascending to the brain. Columns are data acquired before (left), during (middle-left), right after (middle-right), and 25 minutes (right) after feeding with a low-concentration caffeine and sucrose solution. Rows are behavioral videography (top), $\Delta F/F$ (middle) and motion-corrected raw (bottom) two-photon calcium imaging data. This animal ex-

pressed GCaMP6s and tdTomato, throughout the nervous system.

Supplementary Movie 12: Repeatedly recording thoracic cervical connective neural activity before, during, right after, and long after feeding with a high-concentration caffeine and sucrose solution. Two-photon imaging of a cross-section of the thoracic cervical connective including neurons descending from and ascending to the brain. Columns are data acquired before (left), during (middle-left), right after (middle-right), and more than 25 minutes (right) after feeding with a high-concentration caffeine and sucrose solution. Rows are behavioral videography (top), $\Delta F/F$ (middle) and motion-corrected raw (bottom) two-photon calcium imaging data. This animal expressed GCaMP6s and tdTomato, throughout the nervous system.

Supplementary Movie 13: Neural activity waves following high-concentration caffeine ingestion. Two-photon imaging of a cross-section of the thoracic cervical connective including neurons descending from and ascending to the brain. Columns are different occurrences of neural activity waves observed across three animals (here flies '1-3' are flies '7-9' in Supplementary Figure 13) more than 25 minutes after feeding with a sucrose and high-concentration caffeine solution. The second wave of fly 1 occurred in a later trial that is not included in Supplementary Figure 13). Rows are behavioral videography (top), $\Delta F/F$ (middle) and motion-corrected raw (bottom) two-photon calcium imaging data. These animals expressed GCaMP6s and tdTomato, throughout the nervous system.
