# Supplementary material for: Microengineered devices enable long-term imaging of the ventral nerve cord in behaving adult Drosophila
Source: Nat Commun. 2022 Aug 25;13:5006. doi: 10.1038/s41467-022-32571-y (PMC9411199; doi:10.1038/s41467-022-32571-y)
Supplement: Supplementary file 16 — Reporting Summary [file 41467_2022_32571_MOESM16_ESM.pdf]

nature portfolio

Corresponding author(s): Pavan Ramdya, Selman Sakar

Last updated by author(s): Jul 14, 2022

Reporting Summary

Nature Portfolio wishes to improve the reproducibility of the work that we publish. This form provides structure for consistency and transparency in reporting. For further information on Nature Portfolio policies, see our Editorial Policies and the Editorial Policy Checklist.

Statistics

For all statistical analyses, confirm that the following items are present in the figure legend, table legend, main text, or Methods section.

n/a	Confirmed	
☐	☒	The exact sample size (*n*) for each experimental group/condition, given as a discrete number and unit of measurement
☐	☒	A statement on whether measurements were taken from distinct samples or whether the same sample was measured repeatedly
☐	☒	The statistical test(s) used AND whether they are one- or two-sided<br>*Only common tests should be described solely by name; describe more complex techniques in the Methods section.*
☒	☐	A description of all covariates tested
☐	☒	A description of any assumptions or corrections, such as tests of normality and adjustment for multiple comparisons
☐	☒	A full description of the statistical parameters including central tendency (e.g. means) or other basic estimates (e.g. regression coefficient) AND variation (e.g. standard deviation) or associated estimates of uncertainty (e.g. confidence intervals)
☐	☒	For null hypothesis testing, the test statistic (e.g. *F*, *t*, *r*) with confidence intervals, effect sizes, degrees of freedom and *P* value noted<br>*Give P values as exact values whenever suitable.*
☒	☐	For Bayesian analysis, information on the choice of priors and Markov chain Monte Carlo settings
☒	☐	For hierarchical and complex designs, identification of the appropriate level for tests and full reporting of outcomes
☐	☒	Estimates of effect sizes (e.g. Cohen's *d*, Pearson's *r*), indicating how they were calculated

Our web collection on statistics for biologists contains articles on many of the points above.

Software and code

Policy information about availability of computer code

Data collection	Two-photon microscope images were acquired using ThorImage 3.2 software. Data synchronization was performed using ThorSync 3.2 software. Custom Python code were used to acquire behavioral videos. Zen 2011 14.0 software was used to acquire confocal images. SolidWorks 2021 was used to generate CAD.
Data analysis	Data analysis code was written in Python 3 and is available in a GitHub repository: https://github.com/NeLy-EPFL/Long-Term-Imaging-VNC-Drosophila.

For manuscripts utilizing custom algorithms or software that are central to the research but not yet described in published literature, software must be made available to editors and reviewers. We strongly encourage code deposition in a community repository (e.g. GitHub). See the Nature Portfolio guidelines for submitting code & software for further information.

Data

Policy information about availability of data

All manuscripts must include a data availability statement. This statement should provide the following information, where applicable:

- Accession codes, unique identifiers, or web links for publicly available datasets
- A description of any restrictions on data availability
- For clinical datasets or third party data, please ensure that the statement adheres to our policy

All data are available at: https://dataverse.harvard.edu/dataverse/long_term_imaging_vnc_drosophila

Field-specific reporting

Please select the one below that is the best fit for your research. If you are not sure, read the appropriate sections before making your selection.

- [x] Life sciences
- [ ] Behavioural & social sciences
- [ ] Ecological, evolutionary & environmental sciences

For a reference copy of the document with all sections, see nature.com/documents/nr-reporting-summary-flat.pdf

Life sciences study design

All studies must disclose on these points even when the disclosure is negative.

Sample size	The sample size was chosen to be consistent with other studies in the field. For studies of behavior in female flies, 120 flies were used, divided into 3 groups of 40 flies each. For studies of behavior in male flies, 2 groups of 15 flies were used. For the limb amputation experiment, 2 groups of 5 flies were used. For the caffeine ingestion experiment, 3 groups of 3 flies each were used (1 experimental group and 2 control groups).
Data exclusions	In optogenetic behavior experiments, flies that touched one of the walls for more than 0.3 s during optogenetic stimulation were excluded from data analysis. Flies that were on their backs during optogenetic stimulation were also excluded. One trial was excluded for a fly fed the low concentration caffeine solution because one region of the cervical connective was out of the field-of-view for a few frames, This made it difficult to motion correct the imaging data.
Replication	Each sample was considered a replicate in our study. In our behavior experiment, we have n=40 replicates per group. In the limb amputation experiment, n=5 replicates per group and in the caffeine ingestion experiment, n=3 replicates per group.
Randomization	No randomization was performed because we report recordings of the same animals over days.
Blinding	The experiments presented in the study act as proof-of-concepts experiments to validate long-term imaging techniques. Although experiments were not blinded, several investigators performed data analysis to reduce bias.

Reporting for specific materials, systems and methods

We require information from authors about some types of materials, experimental systems and methods used in many studies. Here, indicate whether each material, system or method listed is relevant to your study. If you are not sure if a list item applies to your research, read the appropriate section before selecting a response.

Materials & experimental systems

n/a	Involved in the study	
[ ]	[x]	Antibodies
[x]	[ ]	Eukaryotic cell lines
[x]	[ ]	Palaeontology and archaeology
[ ]	[x]	Animals and other organisms
[x]	[ ]	Human research participants
[x]	[ ]	Clinical data
[x]	[ ]	Dual use research of concern

Methods

n/a	Involved in the study	
[x]	[ ]	ChIP-seq
[x]	[ ]	Flow cytometry
[x]	[ ]	MRI-based neuroimaging

Antibodies

Antibodies used	- Mouse Anti-nc82, Bruchpilot (nc82, DSHB)
Validation	All antibodies were validated by the suppliers. No additional validation was performed in the laboratory.<br>- Mouse Anti-nc82 supplier website - https://dshb.biology.uiowa.edu/nc82

Animals and other organisms

Policy information about studies involving animals; ARRIVE guidelines recommended for reporting animal research

Laboratory animals	The following transgenic Drosophila melanogaster were used in this study:<br>female flies (implanted at 4-6dpe and studied for up to 30 days post implantation) Act88F:Rpr/+;GMR57C10-Gal4/UAS-CD4:tdGFP;+ ,<br>female flies (implanted at 5 dpe and studied over their entire lifespan) UAS-CsChrimson/Act88F:Rpr; VT50660.p65AD(attp40) / +;VT44845.GAL4DBD(attp2) /+,<br>female flies (implanted at 1 dpe and studied for 15 days post implantation) Act88F-Rpr/+; iav-GAL4/UAS-GFP;+,<br>female flies (studied at 5 dpe) Act88F:Rpr/+;GMR57C10-Gal4/UAS-GCaMP6f;UAS-TdTomato/+,<br>female flies (implanted at 3 dpe and studied for 5 more days) Act88F-Rpr/+; GMR22C05-AD-spGal4 / UAS-GCaMP6f; GMR56G08-

	DBD-spGal4 / UAS-tdTomato,<br>male flies (implanted at 1 dpe and kept for 20 days after implantation) Act88F-Rpr; UAS-GFP; +/+.
Wild animals	No wild animals were used.
Field-collected samples	No field-collected samples were used.
Ethics oversight	Ethics oversight was performed by EPFL and the Swiss government.

Note that full information on the approval of the study protocol must also be provided in the manuscript.
